# Collaborative Assessment and Management of Suicidality (CAMS) compared to enhanced treatment as usual (E-TAU) for suicidal patients in an inpatient setting: study protocol for a randomized controlled trial

**DOI:** 10.1186/s12888-020-02589-x

**Published:** 2020-04-22

**Authors:** Miriam Santel, Thomas Beblo, Frank Neuner, Michaela Berg, Kristina Hennig-Fast, David A. Jobes, Martin Driessen

**Affiliations:** 1 Clinic of Psychiatry and Psychotherapy, Ev. Hospital Bethel, Bielefeld, Germany; 2 Department of Clinical Psychology and Psychotherapy, Bielefeld University, Bielefeld, Germany; 3 Department of Psychology, The Catholic University of America, Washington, DC USA

**Keywords:** Randomized controlled trial, Suicidality, Suicidal patients, Treatment, CAMS, Collaborative approach, Suicide prevention

## Abstract

**Background:**

The Collaborative Assessment and Management of Suicidality (CAMS) is a therapeutic framework that has been shown to reduce suicidal ideation and overall symptom distress. CAMS has not been previously evaluated in a standard acute inpatient mental health care setting with only short treatment times for suicidal patients. In this randomized controlled trial (RCT) we are investigating whether CAMS is more effective than Enhanced-Treatment as Usual (E-TAU) in reducing suicidal thoughts as primary outcome variable. We are also investigating depressive symptoms, general symptom relief, and the quality of the therapeutic alliance as secondary outcomes.

**Methods/Design:**

This RCT is designed as a single-center, two-armed, parallel group observer-blinded clinical effectiveness investigation. We are recruiting and randomizing 60 participants with different diagnoses, who are admitted as inpatients because of acute suicidal thoughts or behaviors into the Clinic for Psychiatry and Psychotherapy, Ev. Hospital Bethel in Bielefeld, Germany. The duration of treatment will vary depending on patients’ needs and clinical assessments ranging between 10 and 40 days. Patients are assessed four times, at admission, discharge, 1 month, and 5 months post-discharge. The primary outcome measure is the Beck Scale for Suicide Ideation. Other outcome measures are administered as assessment timepoints including severity of psychiatric symptoms, depression, reasons for living, and therapeutic relationship.

**Discussion:**

This effectiveness study is being conducted on an acute ward in a psychiatric clinic where patients have multiple problems and diagnoses. Treatment is somewhat limited, and therapists have a large caseloads. The results of this study can thus be generalizable to a typical inpatient psychiatric hospital settings.

**Trial registration:**

This clinical trial has been retrospectively registered with the German Clinical Trials Register; registration code/ DRKS-ID: DRKS00013727 (on January 12, 2018). In addition, the study was also registered with the International Clinical Trials Registry Platform of the World Health Organization (identical registration code). Registry Name: „Evaluation von CAMS versus TAU bei suizidalen Patienten – Ein stationärer RCT“.

## Background

Approximately 10,000 people die by suicide in Germany each year, with approximately 28 suicides per day or 1 suicide every 52 min [1]. More than 100,000 people attempt suicide every year in Germany. Over 90% of suicidal cases are associated with mental illness and/or acute crises and suicidality omnipresent within inpatient psychiatric settings [2, 3]. Nevertheless, there are surprisingly few empirically evaluated interventions and guidelines for the actual treatment of suicidal patients [4, 5], particularly within inpatient psychiatric settings.

Traditional treatment approaches for suicidal risk have typically targeted underlying mental disorders with limited proven effectiveness whereas a handful of suicide-focused clinical treatments have been shown to be effective for treating suicide risk through randomized controlled trials (RCT’s) [6]. There is extensive RCT support for Dialectical Behavioral Therapy (DBT) with borderline patients [7, 8], Brief Cognitive Behavioral Therapy (BCBT) for military personnel [9], Cognitive Therapy for Suicide Prevention (CT-SP) with suicide attempters [10] as well as Mindfulness-Based Cognitive Behavioral Therapy (MBCT) for chronically depressed outpatients [11]. Due to time, economic and personnel limitations, however, these treatments may be rarely used in routine clinical practice and may only be effective after a certain prohibitively long duration of therapy. In the case of acute risk of suicidal risk, however, immediately effective shorter-term interventions are needed. Leading suicidologists repeatedly have asked, whether treatment focusing on the mental disorder only is an effective way to reduce the risk of suicide [12–14]. Some have proposed that it might be necessary to focus treatment on suicidal risk as an independent syndrome early in care irrespective of the underlying disorder. Indeed, the establishment of research criteria for suicidal behaviour disorders within the DSM-5 reflects a growing appreciation of this idea. Within their systematic review and meta-analysis, Meerwijk et al. note that psychotherapeutic interventions that directly target suicidal thoughts and behaviours are more effective in reducing suicide attempts and suicidal ideation than interventions that only address these factors indirectly [15]. Consequently, within the contemporary suicide treatment research, there is an increasing emphasis to develop effective treatment approaches that quickly and directly engage the issue of suicidal risk independent of psychiatric diagnoses.

Along these lines, the Collaborative Assessment and Management of Suicidality (CAMS) is a treatment framework for suicidal patients that specifically focuses on patient-defined difficulties and challenges that make consider suicide (referred to as “drivers” within the approach) [13]. CAMS is thus a semi-structured therapeutic framework in which the therapist and the patient engage in as assessment and intervention process that is expressly designed to reduce suicidal risk. CAMS emphasizes an active collaboration between the patient and the therapist which is meant to enhance the therapeutic alliance while increasing the patient’s motivation to live. To date, CAMS is supported by a range of clinical trial studies including eight correlational nonrandomized published trials [13] and four published RCT’s [16–19]. So far, results are promising and suggest that CAMS is superior to other approaches and leads to a rapid and sustained reduction of suicidal ideation, overall symptom distress, and related secondary risk factors such as depression and hopelessness. The impact of CAMS on self-harm and suicide attempts is promising but limited thus far [16]. The usefulness of CAMS in longer and private inpatient settings has already been previously demonstrated [20, 21]. Ryberg et al. have shown CAMS to be effective with combined samples of suicidal inpatients and outpatients [19]. But to further confirm and generalize such results to a broader population requires further research. Thus, there is a need for additional randomized controlled trials with comparable treatment doses of CAMS and TAU within an inpatient context of a standard psychiatric sample of a public health system with a short duration of treatment. Moreover, no CAMS clinical trial studies to date have been carried out in German-speaking countries.

## Method

### Aim of the trial

The primary aim of the current study is to investigate whether CAMS reduces suicidal ideation and suicidal behaviours more than Enhanced Treatment As Usual (E-TAU) within a sample of suicidal inpatients. Secondary aims are to investigate the effects of CAMS versus E-TAU on general symptom burden, depression, reasons for living, and the therapeutic relationship. Furthermore, we plan to test the influence of various moderating variables on treatment outcomes; possible moderators will include diagnostic group (borderline personality disorder in particular), number of previous suicide attempts, treatment duration, number of therapeutic sessions, and baseline levels of psychiatric distress (see [22] for more information on previous moderators of CAMS).

### Trial design

In our pragmatic randomized controlled trial at the Clinic of Psychiatry and Psychotherapy, Ev. Hospital Bethel, Germany, CAMS is being compared to E-TAU within an inpatient crisis setting. The trial is designed as a single-center, two-armed, parallel-group, observer-blinded randomized clinical effectiveness trial. Since we assume that about 50% of the patients we initially examine do not meet the inclusion criteria, 20% do not consent to participate in the study and 20% discontinue treatment, we plan to examine 144 patients of the Clinic who are admitted to the ward due to acute suicidal risk (thoughts or behaviors) in order to obtain our target sample size of 60 patients. After screening and randomizing, the study participants will be followed for about 6 months at four checkpoints t_1_, t_2_, t_3,_ t_4_.

**Fig. 1:**
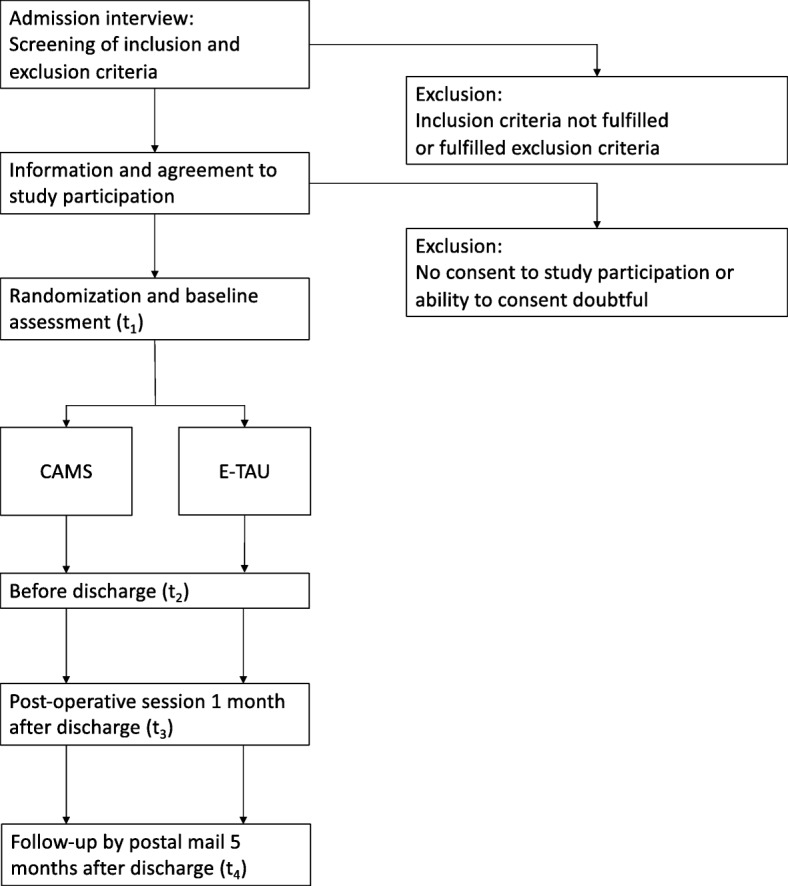
Overview of the examination procedure

Individuals who are screened positive for the trial and who gave their written informed consent to take part in the study are asked to complete a set of questionnaires (t_1_) on the admission day or on the workday following the admission day. Then, the patient is randomized and informed about their group membership and the respective interventions begin on the following day. A second follow up assessment (t_2_) is carried out between 10 and 40 days later at the day before discharge. Another follow-up assessment will be arranged for 4 weeks after discharge (t_3_). Five months after the end of treatment, patients will receive the assessment instruments again by mail (t_4_). Refer to Fig. 1 for a schematic overview of the time flow of the trial and see Appendix 1 for the complete review of the study questionnaires.

## Outcomes of the trial

### Primary outcome

#### Suicidal ideation

#### Beck scale for suicide ideation (BSS)

The primary outcome in the trial is the change in suicidal ideation severity from baseline measure (t_1_) to post assignment (t_2)_ and the follow-up assignments (t_3_ and t_4_). Suicidal Ideation is measured at each assessment by the *German Version of the Beck Scale for Suicide Ideation* (BSS [23];). The Beck Scale for Suicidal Ideation is a self-report instrument for assessing the patient’s severity of suicidal tendencies. For this purpose, 21 statements on a rating scale ranging from 0 to 2 are evaluated as self-assessments and the frequency and seriousness of previous suicide attempts are reported. BSS questions are related to the participant’s wish to live, wish to die, frequency of suicidal ideations, perceived capability to carry out an attempt, extent of actual preparation, and similarly oriented queries. The responses of 19 BSS items are summed up to create an index of suicidal ideation ranging from 0 to 38 with higher scores reflecting greater ideation. The German scale has been found to be a valid and reliable measure of suicidal ideation [24]. The English version of the BSS is widely used in suicide research and has demonstrated predictive validity for suicide attempts and deaths by suicide [25]. The BSS is administered at all study time points.

### Secondary outcomes

#### Depression

#### Beck depression inventory-II (BDI-II)

The BDI-II is a 21-items self-report inventory of depressive symptomatology used to measure depression [26]. It is one of the most widely used research instruments for this purpose and has demonstrated good psychometric properties for use with an inpatient population [27]. Each item is rated on a rating scale from 0 to 3, with higher scores indicating more severe levels of depressive symptoms. Previous studies have reported BDI-II means of 12.75 in a nonclinical student sample [28] and of 21.02 in an inpatient sample [29].

#### General symptom burden

#### Symptom Checklist-18-Mini (SCL-18-Mini)

The SCL-18-Mini is a self-report questionnaire that measures the current extent of the general symptom burden with 18 items [30]. On a Likert scale from 0 (not at all) to 4 (very much), patients should indicate how much they have suffered from various complaints in the last 7 days. Three subscales (somatisation, depression, anxiety) and an overall index for mental distress can be calculated. There are excellent coefficients for the internal consistency (Cronbach’s Alpha); results for convergent, discriminant, and differential validity are satisfactory to good [31].

#### Reasons for living

#### Brief reasons for living inventory (deutsche version) (B-RFL)

The B-RFL is a 12-items form of the *Reasons for Living Invent*ory which functions as a self-report inventory for patients to rate how important each item would be for living if a suicide is contemplated. Inventory Items are rated on Likert Scales of 1 (not at all important as a reason of not killing myself) to 4 (very important as a reason of not killing myself), with higher scores reflecting higher reasons to live [32, 33].

The assessment explores the importance of family and children, religious values, beliefs in the own capabilities, and the value of living in general. It also assesses the fears one may have about what others would think as well as the potential pain involved in a suicidal act which can be important considerations when someone contemplates suicide. The B-RFL has strong psychometric properties and has been shown to be comparable to the Beck Depression Inventory (BDI) or the Beck Hopelessness Scale (BHS) for predicting suicidality [34, 35]. The Brief Reasons for Living Inventory, which has not yet been published in German, was translated from English into German for our study. In order to check and guarantee the linguistic and content-related correctness, a translation-back-translation procedure was conducted with a native speaker.

#### Therapeutic relationship

#### German version of the scale to assess the therapeutic relationship in community mental health care, patient-version (D-STAR-P)

The quality of the therapeutic relationship will be assessed by the patient-version of the D-STAR. The D-STAR-P is a 12-items self-report questionnaire with three subscales: Positive Collaboration, Positive Clinician Input, and Nonsuppurative Clinician Input. Loos et al. (2011) found the psychometric properties to be acceptable [36]. The feasibility and internal consistency of the D-STAR-P was good and there is evidence of good convergent validity.

#### Suicidality

#### Suicide status form (SSF; CAMS condition only)

Administration of the *Suicide Status Form* (initial, interim/tracking, and outcome/disposition versions) is an integral part of the CAMS framework. The SSF is administered within a collaborative engagement process with the patient, as described in the CAMS treatment manual [13]. The SSF is a multifaceted instrument, which is used in the course of the CAMS approach for risk assessment, treatment planning, tracking of risk, as well as for documenting all clinical outcomes. The SSF Core Assessment is made up of five items asking for subjective ratings (0–5) various suicide-related states: psychological pain, stress, agitation, hopelessness, and self-hate (there is also a rating of overall behavioural risk). This set of variables is administered at the start of every CAMS session through the course of care and has strong psychometric validity and reliability with suicidal inpatients [37]. The first three variables (pain, stress, and agitation) are based on Shneidman’s cubic model of psychic pain that lies at the heart of his formulation of the suicidal experiences [38]. The SSF also obtains patient’s sense of hopelessness (based on the work of Aaron Beck), and self-hate which is based on Baumeister’s work conceptualizing suicide as an escape from the pain of self-loathing [39, 40]. The SSF is further divided into additional sections to assess the current risk of suicide and to plan treatment. Responses from the assessment sections of the SSF are used to inform the CAMS Stabilization Plan as well as CAMS treatment goals that focus on patient-defined drivers which make them consider suicide.

### Additional measures

In addition to the outcome measures described above, sociodemographic data (including age, gender, education and current living situation) are recorded for all examination times. Furthermore, a short documentation of medication will take place. This information is gathered in order to describe the characteristics of the study sample.

### Exploratory measures

In order to generate hypotheses for forthcoming studies and to improve our inpatient treatments, the following exploratory outcome is assessed:

#### Components of inpatient treatment

#### Questionnaire to assess the factors subjectively experienced as helpful in the context of inpatient crisis intervention

The questionnaire for the evaluation and assessment of the impact factors of crisis treatment was prepared by us for this study. Patients describe on 14 items how helpful they found different aspects of their inpatient treatment, such as therapeutic sessions, contact with other patients, pharmacotherapy, the setting of the clinic, the help of the social worker etc., and in terms of their overall stabilisation. Inventory items are rated on a Likert Scale from 1 (not helpful at all) to 6 (extremely helpful), with higher scores indicating more satisfaction with the treatment. In addition, patients are asked to assign a letter grade for inpatient treatment and asked to reflect on their health before and after treatment.

We have intentionally limited the number of research measures in order not to overtax patients but believe we will have sufficient data for a meaningful and thorough research endeavour. Completion of the baseline and the follow up questionnaires (t_1_, t_2_, t_3_, t_4_) will take patients approximately 20–30 min each. The test batteries are handed over by the responsible therapist and are administered by the research assistant, who is blind to the patient’s treatment condition.

### Study center

The treatments are provided at the Clinic of Psychiatry and Psychotherapy, Ev. Hospital Bethel in Bielefeld, Germany. The clinic is responsible for the hospital mental health care of the city of Bielefeld with over 340,000 inhabitants. The study is being carried out on a crisis ward with 22 treatment beds; approximately 600 inpatient admissions are performed annually with a mean duration of stay of 13 days (range 1 to 60).

### Study sample

#### Participant inclusion and exclusion criteria

Patients with acute suicidal thoughts or behaviours are included who fluently speak and read the German language and who are capable to agree to participate in the study and provide written informed consent. Most patients are expected to suffer from affective and/or anxiety disorders and/or personality disorders. This study is only treating suicidal inpatients. The consent, legal capacity, and cognitive capacities are clinically evaluated. When participation is clinically judged to be contraindicated for a particular patient, they are excluded from the study.

Patients are also excluded who are chronically suicidal and/or have been treated in an inpatient psychiatric setting for a total of more than 12 weeks within the last 12 months, or who have been admitted more than 6 times during this period, or who live in an assisted living facility. In addition, patients with a psychotic disorder during the last 12 months, an eating disorder with BMI < 16 and/or a current substance dependence are excluded. Substance abuse or previous substance dependence are not exclusion criteria. Patients who are currently suffering from psychotic symptoms as part of an underlying depressive disorder are also excluded, as are patients with developmental disabilities, dementia or an organic condition. Patients are also excluded, who, at the time of admission, have already planned further long-term inpatient or day-clinic treatment to continue after crisis intervention therapy without being discharged in the meantime, as well as those, who have to be treated on our crisis ward for longer than 40 days. However, the duration of the treatment must be at least 10 days in order to ensure that patients receive a sufficient extent of treatment (at least three sessions following the admission interview). The exclusion criteria are established at the time of admission (t_1_) and validated by using the diagnostic interview any few days later. Patients who are committed against their will as per civil legal requirements (PsychKG of the federal state of North Rhine-Westphalia or the nationwide valid law of care) do not take part in the study.

#### Participant retention

In the context of suicidal crises, spontaneous improvements or treatment discontinuations often occur, e.g. because the life conditions of the patients have spontaneously changed. Nevertheless, we make every reasonable effort to retain each randomized intent to treat participant in the study. A telephone appointment between the end of the treatment and the session that takes place 4 weeks later will serve as means to maintain contact with the patients and to optimize retention to post-discharge assessments (t_3_).

#### Subject withdrawal criteria and procedures

In the informed consent procedure, participants are informed from their therapists that they can withdraw their consent from study participation at any time without providing reasons and with no negative consequences. However, clinical staff will ask participants to share their reasons for withdrawal in order to identify any adverse impact of study procedures and to identify any difficulties experienced by subjects during the trial. Withdrawn participants will have the opportunity to continue a regular crisis therapy on the ward if this is clinically indicated.

In addition, participants can be withdrawn from the trial by the investigators, if an exclusion-criteria is found after initial inclusion either during the screening-process or by clinical assessment. For example, a patient who develops psychotic symptoms during the course of treatment may be withdrawn from the study. The participant is then excluded from the trial and continue with a regular treatment in the clinic.

## Procedures

### Recruitment and eligibility screening

The recruitment of the study patients will be carried out according to consecutive admissions to the ward. Patients are referred from the hospital, general practitioners, or from somatic wards after suicide attempts; patients can also self-refer to the clinic setting. When there are clinical indications during the admission interview for inpatient crisis treatment, and the patient fulfils the inclusion criteria, they are verbally informed about the study and are provided with written description of the study at the same or the following day.

#### Diagnostic procedures

With a summary of all available information, the current psychiatric diagnoses are made by at least one psychiatrist and one psychologist at the time of the first admission interview. These diagnoses are double-checked after a few days of care by an independent research assistant with help of a structured clinical interview. The research assistant is a Psychologist (B.Sc.) who has been trained in the diagnostic SCID-interviews. After conducting the clinical diagnostic interview, the research assistant discusses and clinically validates the diagnostic conclusion with the therapist. All diagnostic data will be recorded directly on Case Report Forms and are considered source data.

### Diagnosis

#### Structured clinical interview for DSM-IV Axis I & Axis II

##### SCID- I & SCID-II

The SCID-I is a diagnostic instrument based on diagnostic criteria for Axis I disorders found in the *Diagnostic and Statistical Manual of Mental Disorders*, Fourth Edition (DSM-IV-TR [41–43];). The SCID has been shown to have good reliability with kappa values ranging from .04 to .84; there is an overall mean of .61 for all disorders across a large number of samples. Test-retest reliabilities for disorders in psychiatric patients range from .54 to .84 with a mean of .73. Additionally, the *Structured Clinical Interview for DSM-IV Axis II* was used to identify participants with any personality disorder, because of the higher suicide risk associated with personality disorders, which is particularly true for patients with borderline personality disorder [44]. The SCID interviews will be conducted after the baseline assessment within the first days of study treatment.

### Intelligence

#### Mehrfach-Wortschatz-Intelligenztest (MWT-B)

The Mehrfach-Wortschatz-Intelligenztest [45] is used to measure the general intelligence level according to a simple and reliable scheme using verbal material. Patients are asked in each of the lines to find out if any of the words presented actually exist and to cross it out. In each line, according to the multiple-choice principles, there is one word known in common or scientific language among four fictitious new constructions. The total of 37 items are arranged according to the level of difficulty. The total number of correctly marked lines is compared with the performance of a representative sample of German-speaking adults aged 20 to 64 (*n* = 1952). Then standards (IQ, standard value and percentile rank) can be determined.

**Fig. 2:**
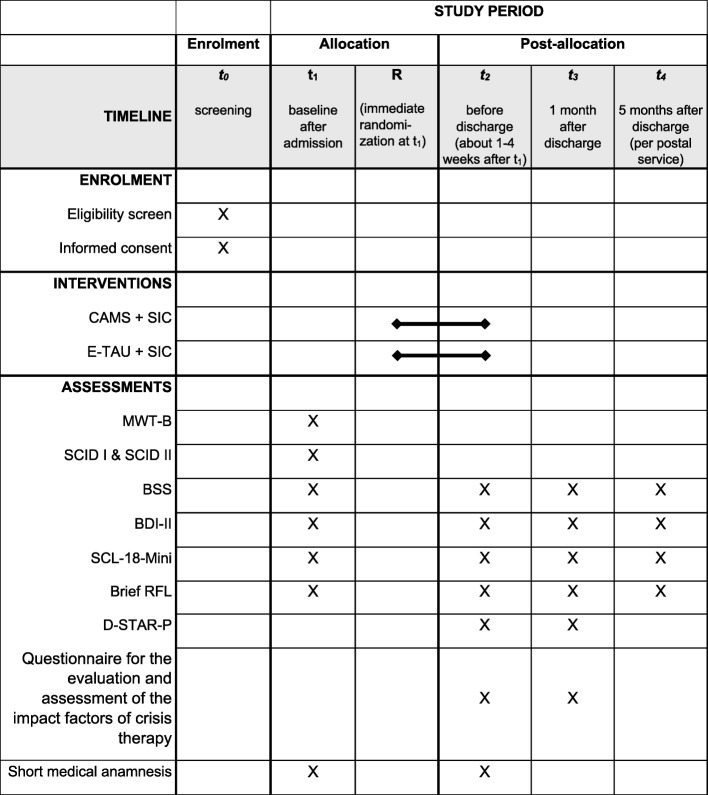
Standard protocol items (SPIRIT) for the clinical trial including enrolment, diagnostic assessments and interventions

See Fig. 2 with all standard protocol items (SPIRIT) for an overview of the procedures of the clinical trial including enrolment, diagnostic and interventions and Appendix 4 for the SPIRIT checklist.

### Interventions

#### Collaborative Assessment and Management of Suicidality (CAMS)

In brief terms, CAMS is a suicide-focused psychotherapeutic approach that targets suicidality as the primary focus of all clinical assessment and treatment. CAMS is organized around the assumption that suicidal thoughts and actions are less a symptom of a mental disorder than an intrapersonal, logical, and often compelling response to psychological stress and suffering. Suicidality can be a great challenge for the therapeutic relationship as it often fosters an opposing dynamic (patient versus therapist) over the struggle to determine whether suicide is an option or not. CAMS is intended to circumvent this adversarial dynamic by enabling the patient and clinician to constructively deal with the patient’s suicidal tendencies within a non-combative and collaborative therapeutic dynamic. Within CAMS, the patient and therapist work together in a focused manner on patient-defined suicidal “drivers” which are those problems that cause the patient to consider suicide. The “collaborative” in CAMS shows itself both content wise and figuratively: Immediately at the beginning of the CAMS intervention, the therapist asks the patient to be allowed to sit next to the patient to fill in the Suicide Status Form (SSF) during the survey (“I want to see it through your eyes”, [13]), so that both can work side by side and together.

CAMS treatment follows a pre-defined format, using the SSF as a multipurpose assessment and treatment planning tool. The continuous monitoring and work on reducing the impact of patient-defined suicidal drivers also creates alternative solutions and perspectives over the course of the sessions ensuring high-quality suicide-focused treatment that also creates detailed medical record documentation. A typical treatment course in our study is characterized by employing two sessions weekly of 30–60 min duration. Every session is initiated by filling out the SSF in a side-by side manner. In the first session, approximately 20 min are used to fill out the first part of the SSF as part of the SSF Core Assessment. At the start of all interim CAMS sessions, approximately 5 minutes is used to complete the SSF Core Assessment (as a repeated measure across CAMS-guided care). A problem-focused treatment plan directly addressing the patient’s suicidal drivers is jointly developed during the first session and routinely evaluated and updated in each following CAMS session. Furthermore, as part of CAMS-guided care, a stabilization plan is developed during the first CAMS session and evaluated and improved during every consecutive session in order to increase the patient’s suicide-related coping skills. Thorough the course of CAMS-guided care stabilization and treating suicidal drivers are central to effective care. Thus, ongoing CAMS care consists of developing adequate coping skills and helping the patient identify and cope with their suicidal drivers more effectively. Within the CAMS framework, suicidal drivers are further divided in two categories; direct and indirect drivers. Direct drivers are thoughts, feelings, or behaviours that increase specific suicidal risk (e.g., being sexually abused as child or break up of a marriage). Indirect drivers are factors that do not produce acute suicidal states but instead increase the vulnerability and the potential activation of direct drivers (e.g., drinking, isolation, insomnia). The CAMS approach does not require any therapeutic orientation and does not prescribe precise interventions as to how exactly suicidal drivers should be treated, which means that the therapist rely on interventions that can treat driver problems (e.g., insight-oriented work, vocational counselling, couples therapy, exposure treatment for trauma).

There are no mandatory homework assignments during a CAMS course of care. The duration of CAMS-guided care is dependent on the treatment progress and varies between patients. The treatment is concluded when the clinician and the patient agree that the acute danger of a suicidal act is eliminated, adaptive coping skills are developed while the patient scores him/herself below 3 on subjective suicidal risk rating (on a 5-point scale) and that they are managing any suicidal thoughts or feelings while remaining behaviorally stable which may trigger discharge from the inpatient setting. In the present RCT, cases who have received between 4 and 9 CAMS sessions including the initial session will be analysed. See Appendix 2 for the complete CAMS Material.

#### Enhanced-treatment as usual (E-TAU)

In addition to the clinic’s standard therapy services, which CAMS patients also receive, patients in the TAU condition also receive supportive, behavioural-therapeutic counselling comparable to the “dose” of CAMS guided care in the experimental arm of the RCT (at least three and up to nine 30–60-min sessions during treatment and one post-operative session). There is no predefined manual for the TAU treatment. In addition to establishing a viable therapeutic relationship and acute relief, diagnostic, psychoeducation and initial therapeutic steps, the planning of further treatment of the underlying mental disorders or life problem will be central. Depending on the current risk situation, the aim is to promote the patient’s safety, to encourage the patient to reflect and to build up confidence and motivation for treatment and the effecting of changes. Depending on the problem areas described by the patient, the practitioners independently determine the focal points and contents of the therapeutic sessions together with the patients. The therapists are free to choose methods and strategies to promote self-control and the use of social support as well as to learn strategies for emotional stabilisation.

**Fig. 3:**
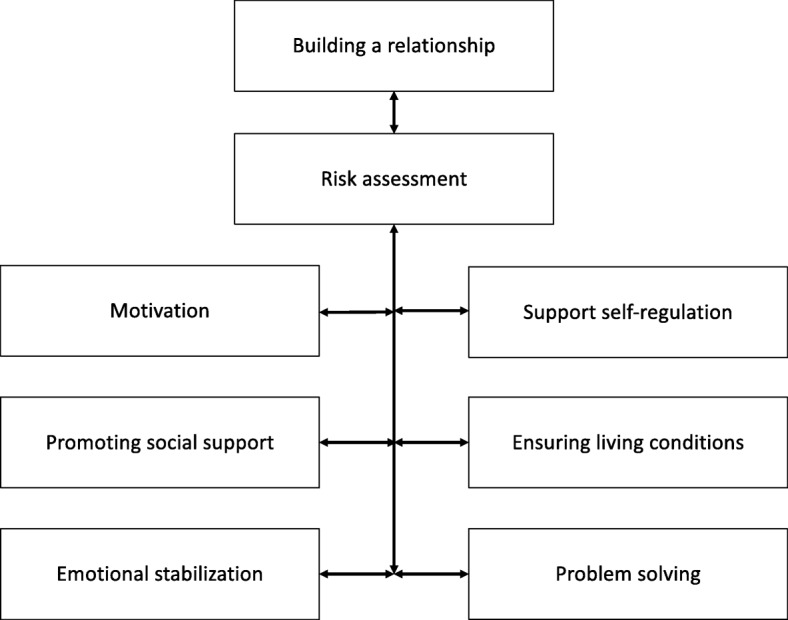
Diagram showing contents of E-TAU treatment

An overview of the various modules of crisis intervention in E-TAU can be found in Fig. 3. To increase experimental internal validity, TAU in this study was “enhanced” (i.e., E-TAU). Patients in E-TAU receive as many treatment sessions as CAMS Patients, approximately 2 per week, so the amount of treatment is comparable in both conditions. Thus, E-TAU is designed to balance and minimize threats to both the internal and external validity of the study.

#### Standard inpatient care (SIC)

SIC contains all non-specific therapy elements that are identical for both intervention groups. In addition to the individual psychotherapeutic sessions described above, there are also occasionally supportive consultations with the nursing staff. Longer individual nursing contacts due to crisis situations will be documented in number and length. A daily morning meeting takes place for all patients. Depending on the patient’s stability and wishes, an individual therapy plan is designed so that the patient can participate in additional offers such as body and movement therapy, Jacobson relaxation, music therapy, occupational therapy and offers from clinical social work and pastoral care. Weekly team meetings serve to exchange information on each individual patient between all the professional groups involved, and weekly visits by a senior physician also take place. The interventions are embedded in the context of a therapeutic environment that offers continuous care, supervision, and plenty of opportunities for spontaneous contact with fellow patients.

### Choice of comparator

The aim of this trial is to test the effectiveness of CAMS for suicidal inpatients on an acute crisis ward. For this purpose, it is necessary to compare CAMS as provided on the ward with the efficacy of the treatment that would be available without this module, i.e. a TAU condition. So far, no data exist regarding the effectiveness of TAU in this understudied population. As described above, TAU in this study was “enhanced” (i.e., E-TAU), which means that the treatment dose is comparable in both conditions, and multiples assessments further improve the patient’s experience, thereby increasing the internal validity of this RCT.

### Medications and treatments permitted/not permitted during the intervention

In both groups, patients receive medication according to their diagnosis and current symptoms, based on the clinical judgement of the senior physician (and following international guidelines). Benzodiazepines and medication on demand can also be used. All medication and changes in medication are recorded in both groups. We expect that medications will be equally represented in both arms as per randomization. In the follow-up assessments, we will determine which follow-up treatments the patients has received (e.g., outpatient psychotherapy or psychiatric treatment as well as any partial care or inpatient treatment).

### Measures taken to minimize/avoid bias, including randomization and blinding

#### Randomization

Patients who meet the inclusion criteria and agree to participate in the study are randomly assigned to one of the two therapy arms either CAMS (36 subjects) or TAU (36 subjects). Randomization is performed by an independent practitioner by throwing the dice immediately after the patients have agreed to participate in the study and returned the completed questionnaires (1–3 = CAMS, 4–6 = E-TAU).

#### Blinding

Due to the behavioral nature of the intervention, neither participants nor therapists and therapy interpreters can be blinded. However, the diagnostic examination is performed by an independent clinician who is blinded for the study group and we tried to keep the group membership confidential from the treatment team and from other patients and study participants, e.g. there was no documentation about the group membership in the patient file. Participants are instructed not to reveal any information related to the group they have been assigned to or regarding the therapeutic process.

#### Trial monitoring

There are regular appointments with the supervisors once a month with the main purpose to observe the progress, the well-being of the patients and to identify problems in conducting the study and to solve problems together. Supervisors have access to all documents (e.g. assessments, audiotapes of therapy sessions, therapy session sheets) to check diagnostic accuracy, consistency of data entry, and adherence to treatment.

#### Treatment fidelity

The therapists in CAMS and TAU are a licensed psychotherapist and a psychologist (Msc.). The assignment to the therapist is consecutive, i.e. the clinician who conducted the admission interview will also be responsible for further treatment. Potential effects of the therapists will be analysed during the evaluation and using post-hoc analyses. We trained CAMS therapists with help of the E-learning training by David Jobes, which both therapists have completed before the start of the study. Additionally, both have worked intensively with the CAMS manual written by David Jobes. The administration of the Suicide Status Form (SSF) was practiced by each therapist with at least 3 patients in a pilot phase prior to the start of the study. This methodology ensured that the therapists internalized both the content and process of CAMS demonstrating sufficient adherence to the CAMS framework. In the event of difficulties, David Jobes provides consultation.

Every fourth treatment session of the therapists is supervised. Each therapy session is audiotaped, and the therapists are rated for adherence according to the CAMS Rating Scale (CRS) by an external evaluator so optimal treatment adherence to CAMS is ensured [46]. The supervisors also monitored the E-TAU sessions using the CRS to ensure and verify that clinicians were not doing CAMS procedures in these sessions thus ensuring experimental fidelity. The CRS is an observer rating scale and consists of three parts and 14 items in total. Part I covers the treatment philosophy, part 2 the clinical/ session framework and part 3 the overall rating. The items were rated on a 6-point scale from 0 = poor to 6 = excellent. The use of the CRS therefore serves to confirm the difference in treatment groups with regard to the fact that the same therapists are treating both groups. We discussed the problem of the CAMS therapists being also the TAU therapists and the question if how the therapy is influenced by the knowledge about the other therapy arm. But in fact, despite the risk of contamination were mitigated by review of videos and use of the CRS. Any experimental between-group differences would provide clear proof of an experimental effect as the clinicians serve as their own control (removing a tremendous source of error variance seen in other RCT’s that use different clinicians in each arm of the trial).

#### Treatment feasibility

The feasibility of the measures is assessed on the basis of dropout rates and the satisfaction with the assigned procedure is also measured. A therapy dropout is operationally defined if the treatment is terminated before the conclusion of at least 3 sessions.

### Measures taken to avoid attrition bias

In order to prevent an attrition bias, we will endeavour to examine patients even after a discontinuation of the study participation (and will conduct our analyses based on intention to treat methodology). In order to estimate the extent of a possible bias, the participants who remain in the study will be compared with those who drop out prematurely. Because we expect some difficulties in collecting follow-up data (for which patients are expected to return to the clinic after discharge), a small expense allowance of 10 Euros for their travel expenses will be offered to reduce any attrition bias due to systematic dropouts.

### Data handling and quality control

Our clinic is responsible for handling of the data generated in our institution according to the General Data Protection Regulation (GDPR). Data storage and transfer will be conducted exclusively in an encrypted manner. Three different kinds of data will be produced at the centers: (1) Personal data including names and contact information, (2), screening, diagnostic, and therapy process data (using pseudonyms for participants), and (3) source data (e.g., audios of assessments and therapy sessions, therapy session sheets). A separate code list with a unique identifier for each participant links the personal data and our use of pseudonyms. The code list is securely stored at the clinic and will be deleted at the end of the project duration. We will transfer the disguised and encrypted data to a secure server accessible only to authorized staff. Checks for completeness will be conducted to ensure data integrity. Statistical analyses will be conducted by independent collaborators. Direct access to trial documents, including source data, is provided for trial-related monitoring and reviews by the ethical committee or regulatory authorities if requested following the guidelines of the GDPR. All individuals who are authorized to perform the aforementioned reviews are bound to confidentiality.

### Statistics

#### Power analysis and sample size calculation

An a priori power analysis by using G*Power was carried out to determine the sample size [47]. On the basis of the available literature, we expect an average effect size (comparison of CAMS versus TAU with regard to the reduction of suicidal tendencies) of Cohen’s d = 1. Here we refer to the study by Ellis (2015), which is rather comparable [21]. The author reports a pre-post effect size of d = 1.72 for CAMS (measured by the BSS) and of d = 0.71 for TAU. Although the interaction effect was not reported we assume it to be about d = 1.

Taking into account a probability of α error = 5% (one-sided) and a power of 1 - β = 80%, the total sample size of 48 participants, i.e. 24 per group. For compensating participants who withdraw their consent we plan 30 subjects per group. Assuming that after the first diagnostic examination 50% are not eligible for inclusion into the study due to non-existent inclusion criteria or exclusion criteria and 20% discontinue treatment in the course of the study or are excluded due to further inpatient treatment, it is to be expected, that 144 patients have to be interviewed, of whose 72 patients (36 per group) are randomly assigned to CAMS intervention (36 subjects) and a TAU (36 subjects), respectively, in order to achieve the targeted number of *N* = 2 × 30 in the intent to treat sample.

### Data analyses

#### Statistical analyses

The analysis of this study will be calculated as a mixed-effects model with the BSS total symptom score as the outcome variable. Mixed models are especially suited for longitudinal studies as they can deal with a correlated data structure and can analyze all cases and data points, even if missing values occur in the course of the trial. In detail, participants will be modeled as a random factor, while time and intervention (CAMS vs. TAU) as well their interaction will be modeled as fixed factors. The hypothesis that CAMS is superior to TAU in the treatment of suicidal patients will be evaluated by the significance test of the interaction effect time × intervention.

In case of a significant interaction effect, two planned general linear hypotheses will be calculated as post-hoc tests for linear mixed effect models in order to test between-group differences at t_2_ and t_3_. *P*-values will be adjusted for multiple comparisons following the Holm procedure.

We will perform an intention-to-treat analyses, that is, all trial participants will be analyzed as randomized, even if they discontinue treatment or are unavailable for one or both of the follow-up interviews. The between-group effect size (Cohen’s d) will be calculated at each follow-up assessment (t_2_ and t_3_).

Continuous secondary outcome measures (BDI-II, SCL-18-Mini, B-RFL, D-STAR-P) are analyzed in the same way. In absence of a valid cut-off score for clinically significant change or treatment response of the BSS, the number of subjects with clinically significant improvement as well as worsening based on the reliable change index (RCI) will be compared between groups using χ2 –tests or Fisher’s exact tests. For this purpose, the RCI will be calculated based on the pre-treatment scores of the study sample. We will use two-tailed tests for statistical significance with alpha set at *P* < 0.05. Effect strength will be determined with Cohen’s d. All calculations will be performed using SPSS 20.0 computer software [48].

We also investigate whether there is a correlation between the number of sessions and the effect of the therapeutic treatment using a correlation analysis.

Interim analyses before the completion of the investigations are not intended. Any potential deviations from the original statistical plan as defined in the study protocol will be described in protocol amendments as well as in the final study report.

### Dissemination policy

#### Dissemination of trial results

The trial results will be disseminated to the scientific community by publications in international peer-reviewed journals. The results regarding the primary outcome of the trial will be published regardless of the direction and statistical significance of the effect.

In addition, we expect that this trial can inform the regular health care system (clinics, psychotherapists in outpatient clinics) about the effect of short-term therapy for suicidal patients and improve the quality of care. As there is a lack of methodological sound RCTs for suicidal inpatients we expect that this trial could have a major impact on the guidelines for the treatment of suicidal patients.

### Safety and ethical aspects of the trial

#### Safety

CAMS previously has been shown to be an effective treatment model. In a pilot study from 2012, Ellis and colleagues found that CAMS was successfully implemented and accepted by both patients and clinicians within the frameworks of an inpatient settings and Ryberg and colleagues (2019) showed that CAMS was successfully implemented within a very broad psychiatric sample with a high symptom load which is probably comparable to our sample [19, 20]. On this basis we expect that CAMS most probably will be acceptable and feasible to the patients and therapists within our clinic. Psychological stress caused by the examination of the causes of suicidal tendencies within the treatment is possible. Negative emotions will occur in all suicidal patients, this is immanent to the symptoms and treatment situation. The team at the ward, on which the study is conducted, is specialized in the treatment of this group of patients. In the event of an increase in psychological stress, patients in emergency situations have nursing staff on the ward and doctors on duty outside regular working hours available at all times for supportive interventions. In an emergency, safety measures can also be initiated in an inpatient context. Patients would be excluded if therapists were given the impression that CAMS or E-TAU treatment is detrimental to the patient.

In the case of suicides in the course of treatment and study participation that may occur in such a high-risk group, these are documented by the therapists.

#### Ethics

The trial has been approved by the Ethical Committee of the Medical Department of the University of Münster in Germany on 6 March 2017. Research on a vulnerable group of patients requires several ethical considerations to be discussed and addressed, especially when it comes to suicidal patients, who are excluded from most treatment studies. The major problem in the field of suicide treatment is that most clinically administered treatments are not supported by randomized controlled trial investigations (the gold standard for understanding the causal impact of a treatment).

If we look at the results of previous CAMS clinical trials, there is no reason to believe that CAMS will have a more negative effect than TAU, rather the opposite is expected. The main intention of the study is to evaluate whether CAMS is more effective than E-TAU. If our hypotheses are supported, our goal is to contribute to the use of CAMS for future inpatients within our own and other clinics. It is important to stress that all suicidal patients will receive treatment during the study, whether this is CAMS or E-TAU. Patients in the study receive twice as many therapeutic consultations as it is assured to other patients on the ward who do not participate in the study. The increased amount of therapeutic consultations in both conditions should motivate patients to participate in the study and ensure that they receive a sufficient number of treatment sessions even in the short treatment periods to make a valid comparison between CAMS and TAU possible and appropriate.

We will not offer a control group who receives no treatment or postponed treatment. Participation is voluntary, and the patients are informed of their possibility to withdraw from the study at any time with no penalty. The patients attending the project are not offered any remuneration, such as payments of gifts for their participation in the trial. Therefore, we expect little to no other motives for participation, except general openness, and hope for an improving care for suicidal inpatients.

#### Data protection and security

During the trial, sensitive data on the mental health status will be collected in the assessments on the Case Report Forms. A code is used as a surrogate for all case report forms; the name of the participant will be replaced by a code. A separate code list will be maintained on paper, linking the participant codes with the name and contact information (for the purpose of contacting participants for the follow-up assessments). This code list will be always locked securely and stored separately from the study records. Furthermore, other source data (e.g. therapy session sheets, audio cassettes of therapy sessions), which cannot be identified by codes, are of a sensitive nature, as they could reveal the identity of the participant. All participant information will be stored in locked filing cabinets. Study records which contain names (e.g., informed consent forms, contact sheets) as well as data which might allow the identification of the participant (e.g., audiotapes) will be stored separately from the Case Report Forms (both physically and electronically).

## Discussion

For our study, we decided to focus on effectiveness of CAMS. As a result, pragmatic considerations guided our methodological decisions for the development and implementation of the study. Within effectiveness studies the “real world” use of an intervention is investigated, and this helps ascertain whether an intervention can work within normal practice. The patients seeking our help in the mental health system often have multiple problems. By making ongoing suicidality the main inclusion criteria and trying to keep the inclusion criteria very broad, we expand the generalizability of the trial results. To enhance the feasibility of this RCT, we use a rather short test battery.

The trial is a single centre trial and can therefore be considered as a pilot trial for multicentre trials in the future. In order to obtain external validity, we need multicentre trials.

In this study we would like to investigate first whether CAMS is superior to TAU treatment and effective in an acute inpatient setting. If this is the case, we would like to investigate the underlying effect mechanisms of CAMS in further studies.

However, we expect some challenges during the implementation of the study. The main concerns are associated with the setting and recruitment. The ward where the study is carried out is an acute crisis ward with numerous very short treatments and a great deal of fluctuation in the patient population which can create some degree of disquiet within the milieu and the additional work for the therapists in the study in everyday life difficult.

Because the inclusion criteria are very broad, we will invite as many patients as possible to participate in the study. There is no preselection. Randomization should reduce the risk of a selection bias and various sources of systematic error. We will also include patients with longer histories of inpatient psychiatric treatments who seem to express suicidal thoughts as “crying for help” in order to receive support, which is sometimes difficult to distinguish from genuine suicidal tendencies. These patients are generally regarded as very difficult to treat. However, we will also include these patients in the study to see if the treatment supports these patients as well. The inclusion of these “chronic” patients may, of course, reduce the effects of the study.

Due to the multiple illnesses of the patients in our clinic, we expect in particular many patients to have personality disorders and special needs and expectations in the treatment, which may cause problems during the treatment and impact adherence to the manual or to treatment dropouts.

And finally, it should be noted that the targeted sample size is quite small, so there must be a large effect to detect differences between conditions.

### Trial status

Protocol identifying number: 0.3

Revision history

**Table:** 

Version	Author	Date	Reason for Revision
0.1	M. Santel	1 March 2019	1st draft version sent to participating authors for review
0.2	M. Santel	24 May 2019	2nd draft version with integrated feedback from all co-authors sent to all co-authors again
1.1	M.Santel	29 May 2019	1st version sent for review
1.2	M.Santel	March 2020	2 ^nd^ revised version

Date: 29 May 2019

The trial opened by recruiting the first patient on 8 March 2017 and is still recruiting. Recruitment is terminated when the targeted number of 30 patients in each treatment group has been reached. Recruitment is expected to be completed in autumn 2019.

## Supplementary information

**Additional file 1:.** Appendix 1 Fragebögen zur CAMS-Studie zur Behandlung suizidaler Krisenpatienten – Complete questionnaires for the CAMS study

**Additional file 2:.** Appendix 2 Suicide Status Forms – Complete CAMS Material

**Additional file 3:.** Appendix 3a Einverständniserklärung zur Teilnahme an der Studie – Informed Consent Form

**Additional file 4:.** Appendix 3b Informationen zur Studie – Patient information

**Additional file 5:.** Appendix 4 SPIRIT checklist – SPIRIT checklist

**Additional file 6:.** Appendix 5a Ethikkommission; Beratung und Bewertung – Consent of the Ethical Committee: Original Document in German

**Additional file 7:.** Appendix 5b Ethikkommission; Consulting and Evaluation – Consent of the Ethical Committee: English translation

## Data Availability

The datasets generated during the current study are not publicly available because data collection is still ongoing. Data will be available on reasonable request by the corresponding author.

## Abbreviations

BCBT: Brief Cognitive Behavioral Therapy
BDI-II: Beck-Depression-Inventory-II
BSS: Becks Scale for Suicidal Ideation
B-RFL: Brief Reasons for Living Inventory
CAMS: Collaborative Assessment and Management of Suicidality
CBT: Cognitive Behavioral Therapy
CRS: CAMS Rating Scale
CT-SP: Cognitive Therapy for Suicide Prevention
DBT: Dialectical Behavior Therapy
DSM: Diagnostic and Statistical Manual of Mental Disorders
D-STAR: German Version of the Scale to assess the Therapeutic Relationship in Community Mental Health Care
E-TAU: Enhanced Treatment As Usual
EvKB: Evangelical Hospital Bethel
GDPR: General Data Protection Regulation
MBCT: Mindfulness-based Cognitive Behavioral Therapy
MWT-B: Mehrfach-Wortschatz-Intelligenztest
RCI: Reliable Change Index
RCT: Randomized Controlled Trial
SCID-I & SCID-II: Structured Clinical Interview for DSM-IV, Axis I & Axis II
SCL-18-Mini: Short version of the Symptom Checklist
SIC: Standard Inpatient Care
SSF: Suicide Status Form
SPSS: Statistical Package for the Social Sciences
TAU: Treatment As Usual
