# Supplementary material for: Collaborative Assessment and Management of Suicidality (CAMS) compared to enhanced treatment as usual (E-TAU) for suicidal patients in an inpatient setting: study protocol for a randomized controlled trial
Source: BMC Psychiatry. 2020 Apr 22;20:183. doi: 10.1186/s12888-020-02589-x (PMC7178967; doi:10.1186/s12888-020-02589-x)
Supplement: Supplementary file 1 — Additional file 1:. Appendix 1 Fragebögen zur CAMS-Studie zur Behandlung suizidaler Krisenpatienten – Complete questionnaires for the CAMS study [file 12888_2020_2589_MOESM1_ESM.pdf]

Teilnehmer-Code: ________________ Datum: ___________________

Fragebögen zur CAMS-Studie zur Behandlung suizidaler Krisenpatienten

Demographische Daten

Bitte schreiben Sie an keiner Stelle in diesem Fragebogenpaket Ihren Namen auf!

Bitte teilten Sie uns zuerst folgende Informationen mit:

Alter: _________ **Geschlecht:** _________

Familienstand:

( ) ledig, allein lebend ( ) ledig, in einer Partnerschaft ( ) verheiratet

( ) getrennt lebend ( ) geschieden ( ) verwitwet

Höchster Schulabschluss: ___________________________

Beruf: _________________________________

Haben Sie jemals einen Suizidversuch begangen? JA ___ NEIN ___

Wenn JA, wie lange ist Ihr letzter Suizidversuch her? ____________________________________

Wenn JA, wie haben Sie versucht sich das Leben zu nehmen?

____

____

Wenn JA, wie fest entschlossen waren Sie bei Ihrem letzten Suizidversuch darin sich selbst umzubringen?

Nicht sehr entschlossen	Etwas entschlossen	Mittelmäßig entschlossen	Ziemlich entschlossen	Absolut entschlossen

Wie viele Suizidversuche haben Sie in Ihrem Leben bisher unternommen? ____________________

Wie viele stationär-psychiatrische Aufenthalte hat es in ihrem Leben bereits gegeben? ____________

Beck-Suizidgedanken-Skala

<u>Anleitung:</u> Bitte lesen Sie sich jede der folgenden Aussagengruppen aufmerksam durch. Kreuzen Sie für jede Gruppe jeweils die Aussage an, die am besten beschreibt, wie Sie sich während der letzten Woche - heute eingeschlossen- gefühlt haben. Versichern Sie sich, dass Sie alle Aussagen einer Gruppe gelesen haben, bevor Sie sich für eine Aussage entscheiden.

<u>Teil 1</u>

1.
0 Ich habe einen mäßigen bis starken Wunsch zu leben.
1 Ich habe einen schwachen Wunsch zu leben.
2 Ich habe keinen Wunsch zu leben.

2.
0 Ich habe keinen Wunsch zu sterben.
1 Ich habe einen schwachen Wunsch zu sterben.
2 Ich habe einen mäßigen bis starken Wunsch zu sterben.

3.
0 Meine Gründe zu leben überwiegen meine Gründe zu sterben.
1 Meine Gründe zu leben oder zu sterben sind in etwa gleich.
2 Meine Gründe zu sterben überwiegen meine Gründe zu leben.

4.
0 Ich habe nicht den Wunsch, mich umzubringen.
1 Ich habe einen schwachen Wunsch, mich umzubringen.
2 Ich habe einen mittleren bis starken Wunsch, mich umzubringen.

5.
0 Ich würde versuchen, mein Leben zu retten, wenn ich mich in einer lebensbedrohlichen Situation wiederfinden würde.
1 Ich würde es darauf ankommen lassen, weiter zu leben oder zu sterben, wenn ich mich in einer lebensbedrohlichen Situation wiederfinden würde.
2 Ich würde keine notwendigen Schritte einleiten, um den Tod zu vermeiden, wenn ich mich in einer lebensbedrohlichen Situation wiederfinden würde.

Falls Sie sowohl bei Aussagengruppe 4 als auch bei Aussagengruppe 5 die „0“ angekreuzt haben, fahren Sie bitte mit Gruppe 20 fort. Sollten Sie jedoch bei einer der beiden Aussagengruppen eine „1“ oder „2“ angekreuzt haben, dann fahren Sie bitte mit Gruppe 6 fort.

<u>Teil 2</u>

6.
0 Ich habe kurze Phasen, in denen ich darüber nachdenke, mich umzubringen. Diese gehen schnell vorüber.
1 Ich habe Phasen, in denen ich darüber nachdenke, mich umzubringen. Diese dauern einige Zeit an.
2 Ich habe lange Phasen, in denen ich darüber nachdenke, mich umzubringen.

7.
0 Ich denke kaum oder nur gelegentlich daran, mich umzubringen.
1 Ich denke häufig daran, mich umzubringen.
2 Ich denke dauernd daran, mich umzubringen.

8.
0 Ich akzeptiere die Vorstellung, mich umzubringen, nicht.
1 Weder akzeptiere ich die Vorstellung, mich umzubringen, noch lehne ich sie ab.
2 Ich akzeptiere die Vorstellung mich umzubringen.

9.
0 Ich kann mich davon abhalten, Selbstmord zu begehen.
1 Ich bin nicht sicher, dass ich mich davon abhalten kann, Selbstmord zu begehen.
2 Ich kann mich nicht davon abhalten, Selbstmord zu begehen.

10.
0 Meine Familie, Freunde, Religion, die Möglichkeit einer Verletzung durch einen gescheiterten Suizidversuch etc. sind Gründe, warum ich mich nicht umbringen würde.
1 Meine Familie, Freunde, Religion, die Möglichkeit einer Verletzung durch einen gescheiterten Suizidversuch etc. sind Gründe, warum ich mich womöglich nicht umbringen würde.
2 Meine Familie, Freunde, Religion, die Möglichkeit einer Verletzung durch einen gescheiterten Suizidversuch etc. sind keine oder nur geringe Gründe, mich nicht umzubringen.

11. 0 Meine Gründe dafür, Selbstmord begehen zu wollen, zielen hauptsächlich darauf ab, andere Personen zu beeinflussen, zum Beispiel, um mit Personen abzurechnen, Personen glücklicher zu machen, Personen auf mich aufmerksam zu machen etc.
1 Meine Gründe dafür, Selbstmord begehen zu wollen, zielen nicht nur darauf ab, andere Personen zu beeinflussen, sondern stellen auch einen Weg dar, meine Probleme zu lösen.
2 Meine Gründe dafür, Selbstmord begehen zu wollen, liegen hauptsächlich darin, meinen Problemen zu entkommen.

12. 0 Ich habe keinen konkreten Plan, wie ich mich umbringen
1 Ich habe mir verschiedene Wege überlegt, mich umzubringen, aber die Details sind noch nicht ausgearbeitet.
2 Ich habe einen konkreten Plan, wie ich mich umbringe.

13. 0 Ich habe keinen Zugang zu einer Methode oder einer Möglichkeit, Selbstmord zu begehen.
1 Die Methode, die ich für einen Selbstmord verwenden würde, braucht Zeit und es bietet sich mir wirklich keine gute Gelegenheit, diese Methode anzuwenden.
2 Ich habe Zugang oder erwarte, Zugang zu der Methode zu haben, die ich für einen Selbstmord wählen würde, und ich habe die Möglichkeit oder sollte sie haben, die Methode anzuwenden.

14. 0 Ich habe nicht den Mut oder die Fähigkeit einen Selbstmord zu begehen.
1 Ich bin unsicher, ob ich den Mut oder die Fähigkeit habe, Selbstmord zu begehen.
2 Ich habe den Mut und die Fähigkeit, Selbstmord zu begehen.

15. 0 Ich gehe nicht davon aus, einen Selbstmordversuch zu begehen.
1 Ich bin unsicher ob ich einen Selbstmordversuch begehen sollte.
2 Ich bin mir sicher, dass ich einen Selbstmordversuch begehen sollte.

16. 0 Ich habe keine Vorbereitungen getroffen, Selbstmord zu begehen.
1 Ich habe einige Vorbereitungen getroffen, Selbstmord zu begehen.
2 Ich habe meine Vorbereitungen, Selbstmord zu begehen, so gut wie abgeschlossen oder vollendet.

17. 0 Ich habe keinen Abschiedsbrief geschrieben.
1 Ich habe darüber nachgedacht, einen Abschiedsbrief zu schreiben, oder habe angefangen, einen zu schreiben, aber nicht beendet.
2 Ich habe einen Abschiedsbrief geschrieben.

18. 0 Ich habe keine Vorkehrungen dafür getroffen, was passieren wird, nachdem ich Selbstmord begangen habe.
1 Ich habe darüber nachgedacht, einige Vorkehrungen dafür zu treffen, was passieren wird, nachdem ich Selbstmord begangen habe.
2 Ich habe eindeutige Vorkehrungen dafür getroffen, was passieren wird, nachdem ich Selbstmord begangen habe.

19. 0 Ich habe meinen Wunsch, mich umzubringen, nicht vor Leuten verborgen.
1 Ich habe mich zurückgehalten, Leuten davon zu erzählen, dass ich mich umbringen möchte.
2 Ich habe versucht, es zu verbergen, zu verheimlichen oder diesbezüglich zu lügen, dass ich Selbstmord begehen möchte.

20. 0 Ich habe noch nie einen Selbstmordversuch unternommen.
1 Ich habe einmal einen Selbstmordversuch unternommen.
2 Ich habe zweimal oder öfter einen Selbstmordversuch unternommen.

Fahren Sie bitte nur mit der nächsten Aussagengruppe fort, wenn Sie schon einmal versucht haben, einen Selbstmordversuch zu begehen.

21. 0 Mein Wunsch zu sterben war während meines letzten Selbstmordversuchs gering.
1 Mein Wunsch zu sterben war während meines letzten Selbstmordversuchs mäßig.
2 Mein Wunsch zu sterben war während meines letzten Selbstmordversuchs hoch.

BDI II

Anleitung: Dieser Fragebogen enthält 21 Gruppen von Aussagen. Bitte lesen Sie jede dieser Gruppen von Aussagen sorgfältig durch und suchen Sie sich dann in jeder Gruppe **eine Aussage** heraus, die am besten beschreibt, wie Sie sich **in den letzten zwei Wochen, einschließlich heute, gefühlt haben**. Kreuzen Sie die Zahl neben der Aussage an, die Sie sich herausgesucht haben (0, 1, 2 oder 3). Falls in einer Gruppe mehrere Aussagen gleichermaßen auf sie zutreffen, kreuzen Sie die Aussage mit der höheren Zahl an. Achten Sie bitte darauf, dass Sie in jeder Gruppe nicht mehr als eine Aussage ankreuzen, das gilt auch für Gruppe 16 (Veränderung der Schlafgewohnheiten) oder Gruppe 18 (Veränderung des Appetits).

1. Traurigkeit

0 *Ich bin nicht traurig*
1 *Ich bin oft traurig*
2 *Ich bin ständig traurig*
3 *Ich bin so traurig oder unglücklich, dass ich es nicht aushalte*

2. Pessimismus

0 *Ich sehe nicht mutlos in die Zukunft*
1 *Ich sehe mutloser in die Zukunft als sonst*
2 *Ich bin mutlos und erwarte nicht, dass meine Situation besser wird*
3 *Ich glaube, dass meine Zukunft hoffnungslos ist und nur noch schlechter wird*

3. Versagensgefühle

0 *Ich fühle mich nicht als Versager*
1 *Ich habe häufiger Versagensgefühle*
2 *Wenn ich zurückblicke, sehe ich eine Menge Fehlschläge*
3 *Ich habe das Gefühl, als Mensch ein völliger Versager zu sein*

4. Verlust von Freude

0 *Ich kann die Dinge genauso gut genießen wie früher*
1 *Ich kann die Dinge nicht mehr so genießen wie früher*
2 *Dinge, die mir früher Freude gemacht haben, kann ich kaum mehr genießen*
3 *Dinge, die mir früher Freude gemacht haben, kann ich überhaupt nicht mehr genießen*

5. Schuldgefühle

0 *Ich habe keine besonderen Schuldgefühle*
1 *Ich habe oft Schuldgefühle wegen Dingen, die ich getan habe oder hätte tun sollen*
2 *Ich habe die meiste Zeit Schuldgefühle*
3 *Ich habe ständig Schuldgefühle*

6. Bestrafungsgefühle

0 *Ich habe nicht das Gefühl, für etwas bestraft zu sein*
1 *Ich habe das Gefühl, vielleicht bestraft zu werden*
2 *Ich erwarte, bestraft zu werden*
3 *Ich habe das Gefühl, bestraft zu sein*

7. Selbstablehnung

0 *Ich halte von mir genauso viel wie immer*
1 *Ich habe Vertrauen in mich verloren*
2 *Ich bin von mir enttäusch*
3 *Ich lehne mich völlig ab*

8. Selbstvorwürfe

0 *Ich kritisiere oder tadle mich nicht mehr als sonst*
1 *Ich bin mir gegenüber kritischer als sonst*
2 *Ich kritisiere mich für all meine Mängel*
3 *Ich gebe mir die Schuld für alles Schlimme was passiert*

9. Selbstmordgedanken

0 *Ich denke nicht daran, mir etwas anzutun*
1 *Ich denke manchmal an Selbstmord, aber ich würde es nicht tun*
2 *Ich möchte mich am liebsten umbringen*
3 *Ich würde mich umbringen, wenn ich die Gelegenheit dazu hätte*

10. Weinen

0 *Ich weine nicht öfter als sonst*
1 *Ich weine jetzt mehr als früher*
2 *Ich weine beim geringsten Anlass*
3 *Ich möchte gern weinen, aber ich kann nicht*

11. Unruhe

0 *Ich bin nicht unruhiger als sonst*
1 *Ich bin unruhiger als sonst*
2 *Ich bin so unruhig, dass es mir schwer fällt, stillzusitzen*
3 *Ich bin so unruhig, dass ich mich ständig bewegen oder etwas tun muss*

12. Interessenverlust

0 *Ich habe das Interesse an anderen Menschen oder an Tätigkeiten nicht verloren*
1 *Ich habe weniger Interesse an anderen Menschen oder an Dingen als sonst*
2 *Ich habe das Interesse an anderen Menschen oder an Dingen zum größten Teil verloren*
3 *Es fällt mir schwer, mich überhaupt für irgendetwas zu interessieren*

---

13. Entschlussunfähigkeit

0 *Ich bin so entschlussfreudig wie immer*
1 *Es fällt mir schwerer als sonst, Entscheidungen zu treffen*
2 *Es fällt mir sehr viel schwerer als sonst, Entscheidungen zu treffen*
3 *Ich habe Mühe, überhaupt Entscheidungen zu treffen*

14. Wertlosigkeit

0 *Ich fühle mich nicht wertlos*
1 *Ich halte mich für weniger wertvoll und nützlich als sonst*
2 *Verglichen mit anderen Menschen fühle ich mich viel weniger wert*
3 *Ich fühle mich völlig wertlos*

---

15. Energieverlust

0 *Ich habe so viel Energie wie immer*
1 *Ich habe weniger Energie als sonst*
2 *Ich habe so wenig Energie, dass ich kaum noch etwas schaffe*
3 *Ich habe keine Energie mehr, um überhaupt noch etwas zu schaffen*

16. Veränderung der Schlafgewohnheiten

0 *Meine Schlafgewohnheiten haben sich nicht verändert*
1a *Ich schlafe etwas mehr als sonst*
1b *Ich schlafe etwas weniger als sonst*
2a *Ich schlafe viel mehr als sonst*
2b *Ich schlafe viel weniger als sonst*
3a *Ich schlafe fast den ganzen Tag*
3b *Ich wache 1-2 Stunden früher auf als sonst und kann nicht mehr einschlafen*

---

17. Reizbarkeit

0 *Ich bin nicht reizbarer als sonst*
1 *Ich bin reizbarer als sonst*
2 *Ich bin viel reizbarer als sonst*
3 *Ich fühle mich dauernd gereizt*

18. Veränderung des Appetits

0 *Mein Appetit hat sich nicht verändert*
1a *Mein Appetit ist etwas schlechter als sonst*
1b *Mein Appetit ist etwas größer als sonst*
2a *Mein Appetit ist viel schlechter als sonst*
2b *Mein Appetit ist viel größer als sonst*
3a *Ich habe überhaupt keinen Appetit*
3b *Ich habe ständig Heißhunger*

---

19. Konzentrationsschwierigkeiten

0 *Ich kann mich so gut konzentrieren wie immer*
1 *Ich kann mich nicht mehr so gut konzentrieren wie sonst*
2 *Es fällt mir schwer, mich längere Zeit auf irgendetwas zu konzentrieren*
3 *Ich kann mich überhaupt nicht mehr konzentrieren*

20. Ermüdung oder Erschöpfung

0 *Ich fühle mich nicht müder oder erschöpfter als sonst*
1 *Ich werde schneller müde oder erschöpft als sonst*
2 *Für viele Dinge, die ich üblicherweise tue, bin ich zu müde oder erschöpft*
3 *Ich bin so müde oder erschöpft, dass ich fast nichts mehr tun kann*

---

21. Verlust an sexuellem Interesse

0 *Mein Interesse an Sexualität hat sich in letzter Zeit nicht verändert*
1 *Ich interessiere mich weniger für Sexualität als früher*
2 *Ich interessiere mich jetzt viel weniger für Sexualität*
3 *Ich habe das Interesse an Sexualität völlig verloren*

SCL-18-Mini

Sie finden hier eine Liste von Problemen und Beschwerden, die man manchmal hat. Bitte lesen Sie jede Frage einzeln sorgfältig durch und entscheiden Sie, wie stark Sie **während der vergangenen 7 Tage bis heute** durch diese Beschwerden gestört oder bedrängt worden sind. Überlegen Sie bitte nicht erst, welche Antwort, den „besten Eindruck" machen könnte, sondern antworten Sie so, wie es für Sie persönlich zutrifft. Machen Sie bitte hinter jeder Frage nur ein Kreuz bei der für Sie am besten zutreffenden Antwort. Streichen Sie versehentliche Antworten deutlich durch und kreuzen Sie danach die richtige Zahl an. **Bitte beantworten Sie jede Frage!**

Überhaupt nicht	Ein wenig	Ziemlich	Stark	Sehr stark
0	1	2	3	4

Wie sehr litten Sie in den vergangenen sieben Tagen unter…

01.	Ohnmachts-und Schwindelgefühlen	0 - 1 – 2 – 3 – 4
02	dem Gefühl, sich für nichts zu interessieren	0 - 1 – 2 – 3 – 4
03.	Nervosität oder innerem Zittern	0 - 1 – 2 – 3 – 4
04.	Herz- und Brustschmerzen	0 - 1 – 2 – 3 – 4
05	Einsamkeitsgefühlen	0 - 1 – 2 – 3 – 4
06.	dem Gefühl gespannt oder aufgeregt zu sein	0 - 1 – 2 – 3 – 4
07.	Übelkeit oder Magenverstimmungen	0 - 1 – 2 – 3 – 4
08.	Schwermut	0 - 1 – 2 – 3 – 4
09.	plötzlichem Erschrecken ohne Grund	0 - 1 – 2 – 3 – 4
10.	Schwierigkeiten beim Atmen	0 - 1 – 2 – 3 – 4
11.	dem Gefühl wertlos zu sein	0 - 1 – 2 – 3 – 4
12.	Schreck- oder Panikanfällen	0 - 1 – 2 – 3 – 4
13.	Taubheit oder Kribbeln in einzelnen Körperteilen	0 - 1 – 2 – 3 – 4
14.	einem Gefühl der Hoffnungslosigkeit angesichts der Zukunft	0 - 1 – 2 – 3 – 4
15.	so starker Ruhelosigkeit, dass Sie nicht still sitzen können	0 - 1 – 2 – 3 – 4
16.	Schwächegefühl in einzelnen Körperteilen	0 - 1 – 2 – 3 – 4
17.	Gedanken, sich das Leben zu nehmen	0 - 1 – 2 – 3 – 4
18.	Furchtsamkeit	0 - 1 – 2 – 3 – 4

Brief Reasons for Living Inventory – deutsche Version

Kurze Skala zur Erfassung der Gründe für das Leben

Viele Menschen haben schon einmal daran gedacht einen Suizid zu begehen. Andere haben dies nie in Erwägung gezogen. Unabhängig davon ob Sie dies schon einmal erwogen haben oder nicht, sind wir interessiert an den Gründen die Sie abhalten würden einen Suizid zu begehen, wenn Ihnen dieser Gedanke käme oder jemand es Ihnen vorschlagen würde.

Wir würden gerne wissen wie wichtig jeder dieser möglichen Gründe zum aktuellen Zeitpunkt für Sie sein würde als Grund sich **NICHT** umzubringen. Jeder Grund kann **von 1 (überhaupt nicht wichtig) bis 4 (extrem wichtig)** eingeschätzt werden. Wenn einer der Gründe nicht auf Sie zutrifft oder Sie die Aussage nicht für wahr halten, dann ist dieser Grund wahrscheinlich nicht wichtig und Sie sollten die 1 auswählen.

1. Überhaupt nicht wichtig (als ein Grund um mich nicht umzubringen; oder: trifft auf mich nicht zu; oder: Ich glaube überhaupt nicht daran).
2. Nicht sehr wichtig
3. Wichtig
4. Extrem wichtig (als ein Grund um mich nicht umzubringen, ich glaube sehr daran und dieser Grund ist sehr wichtig)

Wenn Sie nie daran gedacht haben oder fest überzeugt sind, dass Sie niemals ernsthaft in Erwägung ziehen sich umzubringen ist es dennoch wichtig, dass Sie jeden Grund bewerten auf der Basis warum ein Suizid für Sie keine Alternative ist oder jemals wäre.

1.	Ich glaube, dass ich einen Sinn im Leben finden kann, einen Grund zu leben.	1 – 2 – 3 – 4
2.	Meine Familie ist von mir abhängig und braucht mich	1 – 2 – 3 – 4
3.	Die Folgen könnten für meine Kinder verletzend/ zerstörerisch sein.	1 – 2 – 3 – 4
4.	Ich habe Angst vor der tatsächlichen „Tat“ mich umzubringen.	1 – 2 – 3 – 4
5.	Ich würde nicht wollen, dass Menschen denken, dass ich keine Kontrolle über mein Leben habe.	1 – 2 – 3 – 4
6.	Meine religiösen Überzeugungen verbieten es	1 – 2 – 3 – 4
7.	Ich möchte nicht sterben	1 – 2 – 3 – 4
8.	Ich liebe und erfreue mich zu sehr an meiner Familie und könnte sie nicht verlassen.	1 – 2 – 3 – 4
9.	Ich möchte zusehen, wie meine Kinder aufwachsen	1 – 2 – 3 – 4
10.	Ich habe Angst vor dem Tod	1 – 2 – 3 – 4
11.	Ich bin beunruhigt was andere von mir denken könnten.	1 – 2 – 3 – 4
12.	Ich halte es moralisch für falsch.	1 – 2 – 3 – 4

Deutsche Version der

Scale to Assess the Therapeutic Relationship in Commiunity Mental Halth Care

Patienten-Version (D-STAR-P)

Anleitung

Im Folgenden finden Sie eine Liste von Aussagen, die Erfahrungen beschreiben, die Ärzte und Patienten haben können. Bitte entscheiden Sie, welche Kategorie am besten auf Ihre Erfahrung mit Ihrem/r behandelnden Arzt/Ärztin zutrifft (0 = nie, 1 = selten, 2 = gelegentlich; 3 = oft; 4 = immer). Kreuzen Sie bitte jeweils die entsprechende Nummer an.

		nie	*selten*	*gelegent-lich*	*oft*	*immer*
		0	1	2	3	4
1.	Mein/e Behandler/in spricht mit mir über meine persönlichen Ziele und Gedanken bezüglich der Behandlung.	○	○	○	○	○
2.	Mein/e Behandler/in und ich sind offen zueinander.	○	○	○	○	○
3.	Mein/e Behandler/in und ich haben eine vertrauensvolle Beziehung.	○	○	○	○	○
4.	Ich glaube, mein/e Behandler/in sagt mir nicht die ganze Wahrheit.	○	○	○	○	○
5.	Mein/e Behandler/in und ich sind ehrlich zueinander.	○	○	○	○	○
6.	Mein/e Behandler/in und ich arbeiten auf gemeinsam vereinbarte Ziele hin.	○	○	○	○	○
7.	Mein/e Behandler/in redet mir ins Gewissen.	○	○	○	○	○
8.	Mein/e Behandler/in und ich sind einer Meinung, welche Veränderungen gut für mich wären.	○	○	○	○	○
9.	Mein/e Behandler/in hat wenig Geduld mit mir.	○	○	○	○	○
10.	Ich glaube, dass mein/e Behandler/in mich mag, egal was ich tue oder sage.	○	○	○	○	○
11.	Wir sind einer Meinung, was die wesentlichen Dinge angeht, an denen ich arbeiten muss.	○	○	○	○	○
12.	Ich glaube, mein/e Behandler/in versteht, was meine persönlichen Erfahrungen für mich bedeuten.	○	○	○	○	○

Fragebogen zur Bewertung der subjektiv als hilfreich erlebten Faktoren im Rahmen einer stationären Krisenintervention

Dieser Fragebogen listet bestimmte Elemente auf, die Patienten im Rahmen unserer Krisenbehandlung als hilfreich empfinden können. Bitte lesen Sie jede Aussage aufmerksam durch und wählen Sie dann eine Nummer, die am besten beschreibt wie **hilfreich** Sie jedes Element für sich empfunden haben.

Nutzen sie die untere Skala und umkreisen Sie die passende Nummer rechts neben der jeweiligen Aussage. Bitte nutzen Sie die gesamte Bandbreite der Auswahlmöglichkeiten, um nicht nur in der Mitte (2, 3, 4, 5) oder nur die Extreme (1, 6) auszuwählen.

Was haben Sie im Rahmen unserer Krisenbehandlung als hilfreich empfunden?

	**1 = überhaupt nicht hilfreich**	**2 = ziemlich wenig hilfreich**	**3 = eher nicht hilfreich**	**4 = eher hilfreich**	**5 = ziemlich hilfreich**	**6 = extrem hilfreich**
1. Das stationäre Setting, die Ruhe und die Sicherheit auf der Station.	1	2	3	4	5	6
2. Die Medikamente, die ich bekommen habe.	1	2	3	4	5	6
3. Die Gespräche und der Kontakt zu meinen Mitpatienten.	1	2	3	4	5	6
4. Das, was ich in der Behandlung gelernt habe.	1	2	3	4	5	6
5. Meine veränderten Lebensumstände.	1	2	3	4	5	6
6. Die therapeutischen Gespräche.	1	2	3	4	5	6
7. Die Hilfe durch den Sozialarbeiter der Station.	1	2	3	4	5	6
8. Die Erfahrung, dass ich mit meinen Schwierigkeiten nicht alleine bin.	1	2	3	4	5	6
9. Die Oberarztvisiten.	1	2	3	4	5	6
10. Die Gespräche und die Unterstützung durch das Pflegepersonal.	1	2	3	4	5	6
11. Ergotherapie und / oder Musiktherapie.	1	2	3	4	5	6
12. Bewegung und / oder Entspannung.	1	2	3	4	5	6
13. Die Gruppenangebote auf der Station	1	2	3	4	5	6
14. Anderes:	1	2	3	4	5	6

Verteilen Sie bitte noch drei Schulnoten von 1 (sehr gut) bis 6 (ungenügend):

Meine **Gesamtnote für die stationäre Behandlung:** ______

Mein Gesamtzustand vor der stationären Behandlung: ______

Mein Gesamtzustand jetzt: ______

Wir freuen uns über persönliche Kommentare zur Behandlung.

____

____

____
