# Supplementary material for: Collaborative Assessment and Management of Suicidality (CAMS) compared to enhanced treatment as usual (E-TAU) for suicidal patients in an inpatient setting: study protocol for a randomized controlled trial
Source: BMC Psychiatry. 2020 Apr 22;20:183. doi: 10.1186/s12888-020-02589-x (PMC7178967; doi:10.1186/s12888-020-02589-x)

Suicide Status Form-II (SSF-II) - Erste Sitzung

Initialen: .... Untersucher: .... Datum: ....

Teil A : PatientIn und UntersucherIn gemeinsam! „I WANT TO SEE IT THROUGH YOUR EYES“

Geben Sie bitte bei jeder Frage an, wie Sie sich gerade jetzt fühlen (Bitte zutreffende Zahl ein kreisen). Ordnen Sie nachher in der linken Spalte die Fragen entsprechend der Wichtigkeit, die Sie ihnen zuordnen. Dabei steht 1 für die wichtigste und 5 für die unwichtigste Frage.

Rang	
....	1) Beurteilen Sie den **psychischen Schmerz** (Gefühl der Verletzung, des Leids, des Elends, nicht jedoch Anspannung und Stress oder körperlichen Schmerz):<br>**niedriger psychischer Schmerz: 1 2 3 4 5 : hoher psychischer Schmerz**<br>Ich finde psychisch am schmerzhaftesten: ....
....	2) Beurteilen Sie das Ausmass des **aktuellen Stresszustandes** (Ihr allgemeines Gefühl, unter Druck zu stehen, von etwas überwältigt zu sein u.ä.):<br>**niedriger innerer Stresszustand: 1 2 3 4 5 : hoher innerer Stresszustand**<br>Für mich ist am meisten mit Stress verbunden: ....
....	3) Beurteilen Sie innere **Spannung und Erregung** (bedrängende Gefühlsinhalte, das Gefühl, Sie müssten irgendetwas – ohne zu wissen was – tun; nicht jedoch Verärgerung, nicht „Verleider“):<br>**niedrige Erregung: 1 2 3 4 5 : hohe Erregung**<br>Ich habe am ehesten das Bedürfnis etwas zu tun,<br>um diesem Erregungszustand ein Ende zu setzen, wenn: ....
....	4) Beurteilen Sie die **Hoffnungslosigkeit** (Ihre Erwartung, dass sich die Dinge nicht bessern, ganz egal, was Sie machen werden):<br>**wenig Hoffnungslosigkeit: 1 2 3 4 5 : viel Hoffnungslosigkeit**<br>Ich bin am hoffnungslosesten in Bezug auf: ....
....	5) Beurteilen Sie die **Selbstentwertung**, den Selbsthass (Ihr allgemeines Gefühl, sich selbst nicht zu mögen, keinen Selbstwert zu haben, sich selbst nicht zu respektieren):<br>**wenig Selbstentwertung: 1 2 3 4 5 : viel Selbstentwertung**<br>Was ich an mir am meisten ablehne, ist: ....
	6) Allgemeine Einschätzung der **Suizidgefährdung**:<br>**extrem niedrig (werde mich nicht umbringen): 1 2 3 4 5 : extrem hoch (werde mich umbringen)**

1) Inwiefern sind Ihre Suizidgedanken abhängig von **Gefühlen und Gedanken über sich selbst**?

Überhaupt nicht 1 2 3 4 5 völlig

2) Inwiefern sind Ihre Suizidgedanken abhängig von **Gefühlen oder Gedanken anderen gegenüber**?

Überhaupt nicht 1 2 3 4 5 völlig

Bitte schreiben Sie im Folgenden möglichst viele Gründe oder Motive auf, warum Sie leben wollen, oder warum Sie sterben wollen. Bitte ordnen Sie dann die einzelnen Gründe entsprechend ihrer Wichtigkeit von 1 – 5 in der Spalte „Rang“.

Rang	**Gründe/Motive, die für das Leben sprechen**	Rang	**Gründe/Motive, die für den Tod sprechen**

Mein Wunsch zu leben, ist: Überhaupt nicht vorhanden: 1 2 3 4 5 6 7 8: ganz besonders stark

Mein Wunsch zu sterben, ist: Überhaupt nicht vorhanden: 1 2 3 4 5 6 7 8: ganz besonders stark

Das, was mir am meisten helfen würde, nicht mehr an Suizid zu denken, wäre ....

....

Teil B (Untersucher)

Ja Nein Suizidplan: Wann: ....

Wo: ....

Wie: .... Zugang zu Suizidmitteln: Ja Nein

Ja Nein Suizidvorbereitungen Beschreibe: ....

Ja Nein Probehandlungen Beschreibe: ....

Ja Nein Suizidanamnese

- Suizidgedanken Beschreibe: ....
  - Häufigkeit .... pro Tag .... pro Woche .... pro Monat
  - Dauer .... Sekunden .... Minuten .... Stunden
- Ein Versuch Beschreibe: ....
- Mehrere Versuche Beschreibe: ....

Ja Nein Aktuelle Suizidabsicht Subjektiv Beschreibe: ....

Objektiv Beschreibe: ....

Ja Nein Impulsivität Subjektiv Beschreibe: ....

Objektiv Beschreibe: ....

Optional auszufüllen

Ja Nein Substanzmissbrauch Beschreibe: ....

Ja Nein Bedeutende Verluste Beschreibe: ....

Ja Nein Soziale Isolation Beschreibe: ....

Ja Nein Beziehungsschwierigkeiten Beschreibe: ....

Ja Nein Gesundheitliche Probleme Beschreibe: ....

Ja Nein Probleme mit dem Gesetz Beschreibe: ....

Teil C (Untersucher): **BEHANDLUNGSPLAN (bezogen auf Teil A& B)**

Problem	Problembeschreibung	Ziele und objektive Kriterien zur Zielerreichung	Interventionen (Art und Häufigkeit)	geschätzte Dauer
1.	*Selbstverletzungsgefahr*	*Sicherheit und Stabilität*	*Stabilisierungsplan erarbeitet* ☐	
2.				
3.				

Ist der Patient / die Patientin mit dem Behandlungsplan einverstanden und zufrieden? JA __ NEIN __
Besteht aktuell akute Suizidalität? JA __ NEIN __

Unterschrift des Patienten Datum Unterschrift des Behandlers Datum

CAMS Suizidstatusform SSF IV (STABILISIERUNGSPLAN)

Möglichkeiten den Zugang zu letalen Mitteln zu reduzieren:

1. ____
2. ____
3. ____

Dinge, die ich tun kann, wenn ich in eine suizidale Krise gerate (vergleichbar mit einer Krisenkarte)

1. ____
2. ____
3. ____
4. ____
5. ____
6. Notfallnummer (z.B. Krisendienst): ____

Menschen, die ich anrufen kann, um nicht allein zu sein oder die ich um Hilfe bitten kann:

1. ____
2. ____
3. ____

Mögliche Barrieren, die vereinbarte Behandlung nicht wahrzunehmen und Lösungen, um die ich mich bemühen werde:

1. ____
2. ____

Teil D (Evaluation des Behandlers nach der Sitzung):

**Suizidalität:**		☐ nein	
☐ ja		☐ möglich	
**Bewusstseinsstörungen:**		☐ keine	
☐ hypervigilant	☐ somnolent	☐ soporös	☐ komatös
☐ Trübung	☐ Einengung	☐ Verschiebung	
**Orientierungsstörungen:**		☐ keine	
☐ zeitlich	☐ örtlich	☐ situativ	☐ zur eigenen Person
**Aufmerksamkeits- und Gedächtnisstörungen:**			☐ keine
☐ Auffassungs-störungen	☐ Konzentrations-störungen	☐ Gedächtnis-störungen*	☐ Merkfähigkeits-störungen
☐ Konfabulationen	☐ Paramnesien	** ggf. MMST durchführen*	
**Formale Denkstörungen:**		☐ keine	
☐ gehemmt	☐ verlangsamt	☐ umständlich	☐ eingeengt
☐ perseverierend	☐ Grübeln	☐ Gedankendrängen	☐ ideenflüchtig
☐ Vorbeireden	☐ gesperrt/ Gedankenabreißen	☐ inkohärent/ zerfahren	☐ Neologismen
**Befürchtungen und Zwänge:**		☐ keine	
☐ Misstrauen	☐ Hypochondrie	☐ Phobien	
☐ Zwangsdenken	☐ Zwangsimpulse	☐ Zwangshandlungen	
**Wahn:**		☐ kein	
☐ Wahnstimmung	☐ Wahn-wahrnehmung	☐ Wahneinfall	☐ Wahngedanken
☐ Systematisierter Wahn	☐ Wahndynamik	☐ Beziehungswahn	☐ Beeinträchtigungs-/ Verfolgungswahn
☐ Eifersuchtswahn	☐ Schuldwahn	☐ Verarmungswahn	☐ Hypochondr. Wahn
☐ Größenwahn		☐ andere Wahninhalte	
**Sinnestäuschungen:**		☐ keine	
☐ Illusionen	☐ Stimmenhören	☐ andere akustische Halluzinationen	☐ optische Halluzinationen
☐ Körperhalluzinationen		☐ Geruchs-/Geschmackshalluzinationen	
**Ich-Störungen:**		☐ keine	
☐ Derealisation	☐ Depersonalisation		
☐ Gedanken-ausbreitung	☐ Gedankenentzug	☐ Gedanken-eingebung	☐ andere Fremd-beeinflussungserlebnisse
**Störungen der Affektivität:**		☐ keine	
☐ ratlos	☐ Gefühl der Gefühllosigkeit	☐ affektarm	☐ Störung der Vitalgefühle
☐ deprimiert*	☐ hoffnungslos	☐ ängstlich	☐ euphorisch
☐ dysphorisch	☐ gereizt	☐ innerlich unruhig	☐ klagsam/jammrig
☐ Insuffizienzgefühle	☐ gesteigertes Selbst-wertgefühl	☐ Schuldgefühle	☐ Verarmungsgefühle
☐ ambivalent	☐ Parathymie	☐ affektlabil	☐ affektinkontinent
☐ affektstarr	** ggf. BDI aushändigen*		
**Antriebs- und psychomotorische Störungen:**			☐ keine
☐ antriebsarm	☐ antriebsgehemmt	☐ antriebsgesteigert	☐ motorisch unruhig
☐ Parakinesen	☐ manieriert/bizarr	☐ theatralisch	☐ mutistisch
☐ logorrhoisch			
**Circadiane Besonderheiten:**		☐ keine	
☐ morgens schlechter		☐ abends besser	
**Andere Störungen:**		☐ keine	
☐ sozialer Rückzug	☐ soz. Umtriebigkeit	☐ Aggressivität	☐ Selbstbeschädigung
☐ Mangel an Krankheitsgefühl	☐ Mangel an Krankheitseinsicht	☐ Ablehnung der Behandlung	☐ pflegebedürftig

Diagnostische Einschätzung / Diagnosen:

____

____

____

Aktuelles Ausmaß der Suizidgefährdung (eins auswählen und erläutern):

- ☐ kein signifikantes Risiko
- ☐ leicht
- ☐ mittel
- ☐ stark
- ☐ extrem

Erklärung: ____

____

____

Fallnotizen (Diagnose, Funktionsfähigkeit, Behandlungsplan, Symptome, Prognose und aktueller Stand):

____

____

____

____

____

Nächster vereinbarter Sitzungstermin: _______________

____

Datum und Unterschrift TherapeutIn

Suicide Status Form-II (SSF-II) – Verlaufssitzung

Initialen: .... Untersucher: .... Datum: ....

Teil A (PatientIn):

Geben Sie bitte bei jeder Frage an, wie Sie sich gerade jetzt fühlen.

1) Beurteilen Sie den **psychischen Schmerz** (Gefühl der Verletzung, des Leids, des Elends, nicht jedoch Anspannung und Stress oder körperlichen Schmerz):
niedriger psychischer Schmerz: 1 2 3 4 5 : hoher psychischer Schmerz

2) Beurteilen Sie das Ausmass des **aktuellen Stresszustandes** (Ihr allgemeines Gefühl, unter Druck zu stehen, von etwas überwältigt zu sein u.ä.):
niedriger innerer Stresszustand: 1 2 3 4 5 : hoher innerer Stresszustand

3) Beurteilen Sie innere **Spannung und Erregung** (bedrängende Gefühlsinhalte, das Gefühl, Sie müssten irgendetwas – ohne zu wissen was – tun; nicht jedoch Verärgerung, nicht „Verleider"):
niedrige Erregung: 1 2 3 4 5 : hohe Erregung

4) Beurteilen Sie die **Hoffnungslosigkeit** (Ihre Erwartung, dass sich die Dinge nicht bessern, ganz egal, was Sie machen werden):
wenig Hoffnungslosigkeit: 1 2 3 4 5 : viel Hoffnungslosigkeit

5) Beurteilen Sie die **Selbstentwertung**, den Selbsthass (Ihr allgemeines Gefühl, sich selbst nicht zu mögen, keinen Selbstwert zu haben, sich selbst nicht zu respektieren):
wenig Selbstentwertung: 1 2 3 4 5 : viel Selbstentwertung

6) Allgemeine Einschätzung der **Suizidgefährdung**:
extrem niedrig (werde mich nicht umbringen): 1 2 3 4 5 : extrem hoch (werde mich umbringen)

Seit der letzten Sitzung: Suizidale Gedanken: Ja Nein Bewältigung dieser Gedanken: Ja Nein
Suizidales Verhalten: Ja Nein

Teil B (Untersucher)

Auflösung der Suizidalität, wenn: aktuelles Suizidrisiko < 3; seit der vorigen Sitzung: kein suizidales Verhalten und effektiver Umgang mit suizidalen Gedanken und Impulsen ☐ 1. Sitzung ☐ 2. Sitzung
Ausfüllen der SSF Ergebnismessung nach 3 nachfolgenden Sitzungen ohne Suizidalität

BEHANDLUNGSPLAN - ÜBERPRÜFUNG

Problem	Problembeschreibung	Ziele und objektive Kriterien zur Zielerreichung	Interventionen (Art und Häufigkeit)	geschätzte Dauer
1.	*Selbstgefährdung*	*Sicherheit und Stabilität*	*Stabilisierungsplan erarbeitet* ☐	
2.				
3.				

Unterschrift des Patienten Datum Unterschrift des Behandlers Datum

Teil C (Evaluation des Behandlers nach der Sitzung):

**Suizidalität:**		☐ nein	
☐ ja		☐ möglich	
**Bewusstseinsstörungen:**		☐ keine	
☐ hypervigilant	☐ somnolent	☐ soporös	☐ komatös
☐ Trübung	☐ Einengung	☐ Verschiebung	
**Orientierungsstörungen:**		☐ keine	
☐ zeitlich	☐ örtlich	☐ situativ	☐ zur eigenen Person
**Aufmerksamkeits- und Gedächtnisstörungen:**			☐ keine
☐ Auffassungs-störungen	☐ Konzentrations-störungen	☐ Gedächtnis-störungen*	☐ Merkfähigkeits-störungen
☐ Konfabulationen	☐ Paramnesien	** ggf. MMST durchführen*	
**Formale Denkstörungen:**		☐ keine	
☐ gehemmt	☐ verlangsamt	☐ umständlich	☐ eingeengt
☐ perseverierend	☐ Grübeln	☐ Gedankendrängen	☐ ideenflüchtig
☐ Vorbeireden	☐ gesperrt/ Gedankenabreißen	☐ inkohärent/ zerfahren	☐ Neologismen
**Befürchtungen und Zwänge:**		☐ keine	
☐ Misstrauen	☐ Hypochondrie	☐ Phobien	
☐ Zwangsdenken	☐ Zwangsimpulse	☐ Zwangshandlungen	
**Wahn:**		☐ kein	
☐ Wahnstimmung	☐ Wahn-wahrnehmung	☐ Wahneinfall	☐ Wahngedanken
☐ Systematisierter Wahn	☐ Wahndynamik	☐ Beziehungswahn	☐ Beeinträchtigungs-/ Verfolgungswahn
☐ Eifersuchtswahn	☐ Schuldwahn	☐ Verarmungswahn	☐ Hypochondr. Wahn
☐ Größenwahn		☐ andere Wahninhalte	
**Sinnestäuschungen:**		☐ keine	
☐ Illusionen	☐ Stimmenhören	☐ andere akustische Halluzinationen	☐ optische Halluzinationen
☐ Körperhalluzinationen		☐ Geruchs-/Geschmackshalluzinationen	
**Ich-Störungen:**		☐ keine	
☐ Derealisation	☐ Depersonalisation		
☐ Gedanken-ausbreitung	☐ Gedankenentzug	☐ Gedanken-eingebung	☐ andere Fremd-beeinflussungserlebnisse
**Störungen der Affektivität:**		☐ keine	
☐ ratlos	☐ Gefühl der Gefühllosigkeit	☐ affektarm	☐ Störung der Vitalgefühle
☐ deprimiert*	☐ hoffnungslos	☐ ängstlich	☐ euphorisch
☐ dysphorisch	☐ gereizt	☐ innerlich unruhig	☐ klagsam/jammrig
☐ Insuffizienzgefühle	☐ gesteigertes Selbst-wertgefühl	☐ Schuldgefühle	☐ Verarmungsgefühle
☐ ambivalent	☐ Parathymie	☐ affektlabil	☐ affektinkontinent
☐ affektstarr	** ggf. BDI aushändigen*		
**Antriebs- und psychomotorische Störungen:**			☐ keine
☐ antriebsarm	☐ antriebsgehemmt	☐ antriebsgesteigert	☐ motorisch unruhig
☐ Parakinesen	☐ manieriert/bizarr	☐ theatralisch	☐ mutistisch
☐ logorrhoisch			
**Circadiane Besonderheiten:**		☐ keine	
☐ morgens schlechter		☐ abends besser	
**Andere Störungen:**		☐ keine	
☐ sozialer Rückzug	☐ soz. Umtriebigkeit	☐ Aggressivität	☐ Selbstbeschädigung
☐ Mangel an Krankheitsgefühl	☐ Mangel an Krankheitseinsicht	☐ Ablehnung der Behandlung	☐ pflegebedürftig

Diagnostische Einschätzung / Diagnosen:

____

____

____

Aktuelles Ausmaß der Suizidgefährdung (eins auswählen und erläutern):

- ☐ kein signifikantes Risiko
- ☐ leicht
- ☐ mittel
- ☐ stark
- ☐ extrem

Erklärung: ____

____

____

Fallnotizen (Diagnose, Funktionsfähigkeit, Behandlungsplan, Symptome, Prognose und aktueller Stand):

____

____

____

____

____

Nächster vereinbarter Sitzungstermin: _______________

____
Datum und Unterschrift TherapeutIn

Suicide Status Form-II (SSF-II) Abschlusssitzung

Initialen: .... Untersucher: .... Datum: ....

Teil A (PatientIn):

Geben Sie bitte bei jeder Frage an, wie Sie sich gerade jetzt fühlen.

1) Beurteilen Sie den **psychischen Schmerz** (Gefühl der Verletzung, des Leids, des Elends, nicht jedoch Anspannung und Stress oder körperlichen Schmerz):<br>**niedriger psychischer Schmerz: 1 2 3 4 5 : hoher psychischer Schmerz**
2) Beurteilen Sie das Ausmass des **aktuellen Stresszustandes** (Ihr allgemeines Gefühl, unter Druck zu stehen, von etwas überwältigt zu sein u.ä.):<br>**niedriger innerer Stresszustand: 1 2 3 4 5 : hoher innerer Stresszustand**
3) Beurteilen Sie innere **Spannung und Erregung** (bedrängende Gefühlsinhalte, das Gefühl, Sie müssten irgendetwas – ohne zu wissen was – tun; nicht jedoch Verärgerung, nicht „Verleider"):<br>**niedrige Erregung: 1 2 3 4 5 : hohe Erregung**
4) Beurteilen Sie die **Hoffnungslosigkeit** (Ihre Erwartung, dass sich die Dinge nicht bessern, ganz egal, was Sie machen werden):<br>**wenig Hoffnungslosigkeit: 1 2 3 4 5 : viel Hoffnungslosigkeit**
5) Beurteilen Sie die **Selbstentwertung**, den Selbsthass (Ihr allgemeines Gefühl, sich selbst nicht zu mögen, keinen Selbstwert zu haben, sich selbst nicht zu respektieren):<br>**wenig Selbstentwertung: 1 2 3 4 5 : viel Selbstentwertung**
6) Allgemeine Einschätzung der **Suizidgefährdung**:<br>**extrem niedrig (werde mich nicht umbringen): 1 2 3 4 5 : extrem hoch (werde mich umbringen)**

Seit der letzten Sitzung: Suizidale Gedanken: Ja Nein Bewältigung dieser Gedanken: Ja Nein

Suizidales Verhalten: Ja Nein

Gab es irgendwelche Aspekte in der Behandlung, die Sie als besonders hilfreich empfunden haben? Wenn ja, bitte beschreiben Sie diese so genau wie möglich.

Was haben Sie im Rahmen der CAMS-Behandlung gelernt, das ihnen helfen kann, falls Sie in der Zukunft suizidal werden sollten?

Teil B (Untersucher)

ERGEBNIS – AKTUELLE SITUATION (Bitte alles zutreffende markieren)

____ ambulante Psychotherapie ____ (teil-) stationäre Weiterbehandlung

____ einvernehmliche Beendigung der Maßnahme ____ Abbruch der Behandlung durch den Patienten

____ Anderes: ____

Nächster vereinbarter Termin: ___________________________

Unterschrift des Patienten Datum Unterschrift des Behandlers Datum

Teil C (Evaluation des Behandlers nach der Sitzung):

**Suizidalität:**		☐ nein	
☐ ja		☐ möglich	
**Bewusstseinsstörungen:**		☐ keine	
☐ hypervigilant	☐ somnolent	☐ soporös	☐ komatös
☐ Trübung	☐ Einengung	☐ Verschiebung	
**Orientierungsstörungen:**		☐ keine	
☐ zeitlich	☐ örtlich	☐ situativ	☐ zur eigenen Person
**Aufmerksamkeits- und Gedächtnisstörungen:**			☐ keine
☐ Auffassungs-störungen	☐ Konzentrations-Störungen	☐ Gedächtnis-störungen*	☐ Merkfähigkeits-störungen
☐ Konfabulationen	☐ Paramnesien	** ggf. MMST durchführen*	
**Formale Denkstörungen:**		☐ keine	
☐ gehemmt	☐ verlangsamt	☐ umständlich	☐ eingeengt
☐ perseverierend	☐ Grübeln	☐ Gedankendrängen	☐ ideenflüchtig
☐ Vorbeireden	☐ gesperrt/ Gedankenabreißen	☐ inkohärent/ zerfahren	☐ Neologismen
**Befürchtungen und Zwänge:**		☐ keine	
☐ Misstrauen	☐ Hypochondrie	☐ Phobien	
☐ Zwangsdenken	☐ Zwangsimpulse	☐ Zwangshandlungen	
**Wahn:**		☐ kein	
☐ Wahnstimmung	☐ Wahn-wahrnehmung	☐ Wahneinfall	☐ Wahngedanken
☐ Systematisierter Wahn	☐ Wahndynamik	☐ Beziehungswahn	☐ Beeinträchtigungs-/ Verfolgungswahn
☐ Eifersuchtswahn	☐ Schuldwahn	☐ Verarmungswahn	☐ Hypochondr. Wahn
☐ Größenwahn		☐ andere Wahninhalte	
**Sinnestäuschungen:**		☐ keine	
☐ Illusionen	☐ Stimmenhören	☐ andere akustische Halluzinationen	☐ optische Halluzinationen
☐ Körperhalluzinationen		☐ Geruchs-/Geschmackshalluzinationen	
**Ich-Störungen:**		☐ keine	
☐ Derealisation	☐ Depersonalisation		
☐ Gedanken-ausbreitung	☐ Gedankenentzug	☐ Gedanken-eingebung	☐ andere Fremd-beeinflussungserlebnisse
**Störungen der Affektivität:**		☐ keine	
☐ ratlos	☐ Gefühl der Gefühllosigkeit	☐ affektarm	☐ Störung der Vitalgefühle
☐ deprimiert*	☐ hoffnungslos	☐ ängstlich	☐ euphorisch
☐ dysphorisch	☐ gereizt	☐ innerlich unruhig	☐ klagsam/jammrig
☐ Insuffizienzgefühle	☐ gesteigertes Selbst-wertgefühl	☐ Schuldgefühle	☐ Verarmungsgefühle
☐ ambivalent	☐ Parathymie	☐ affektlabil	☐ affektinkontinent
☐ affektstarr	** ggf. BDI aushändigen*		
**Antriebs- und psychomotorische Störungen:**			☐ keine
☐ antriebsarm	☐ antriebsgehemmt	☐ antriebsgesteigert	☐ motorisch unruhig
☐ Parakinesen	☐ manieriert/bizarr	☐ theatralisch	☐ mutistisch
☐ logorrhoisch			
**Circadiane Besonderheiten:**		☐ keine	
☐ morgens schlechter		☐ abends besser	
**Andere Störungen:**		☐ keine	
☐ sozialer Rückzug	☐ soz. Umtriebigkeit	☐ Aggressivität	☐ Selbstbeschädigung
☐ Mangel an Krankheitsgefühl	☐ Mangel an Krankheitseinsicht	☐ Ablehnung der Behandlung	☐ pflegebedürftig

Diagnostische Einschätzung / Diagnosen:

____

____

____

Aktuelles Ausmaß der Suizidgefährdung (eins auswählen und erläutern):

- ☐ kein signifikantes Risiko
- ☐ leicht
- ☐ mittel
- ☐ stark
- ☐ extrem

Erklärung: ____________________________________

____

____

Fallnotizen (Diagnose, Funktionsfähigkeit, Behandlungsplan, Symptome, Prognose und aktueller Stand):

____

____

____

____

____

Nächster vereinbarter Sitzungstermin: _______________

____

Datum und Unterschrift TherapeutIn

Das Therapeutische Arbeitsblatt des CAMS

zum Verständnis Ihrer Suizidalität

Datum der Sitzung: ____________________ Sitzungs-Nummer: ____________________

1. Die persönliche Geschichte Ihrer Suizidalität:

Warum sind Sie suizidal? Wie verstehen Sie Ihre Suizidalität? Wie ist Ihre Einstellung zum Suizid? Was ist Ihre persönliche Geschichte?

____

____

____

____

2. Auslöser Ihrer Suizidalität

Problem Nr. 1: ____

Problem Nr. 2: ____

Nun lassen Sie uns herausfinden, welche Faktoren Ihre Suizidalität bedingen und diese auslösen bzw. verstärken. Bitte füllen Sie nur die Abschnitte aus, die für Ihre eigenes Erleben von Suizidalität relevant sind. Ihre Antworten können sich mit Ihren Aussagen überschneiden, die Sie bei der Arbeit mit der Suizid-Status-Form in der ersten Sitzung angegeben haben. Im Behandlungsverlauf können jedoch neue Informationen hinzugefügt werden, um Ihr persönliches Erleben von Suizidalität bestmöglich zu reflektieren.

Was sind die „**direkten Auslöser**“, die unmittelbar dazu führen, dass ich mich suizidal fühle?

Bestimmte Gedanken (z.B. „Es wäre für alle leichter, wenn ich tot wäre“)

____

____

____

____

Bestimmte Gefühle (z.B. „Ich schäme mich einfach so sehr“)

____

____

____

____

Bestimmte Verhaltensweisen (z.B. „Wenn ich den ganzen Tag rumhänge und nichts tue“)

____

____

____

____

Was sind die „indirekten Auslöser“, die dazu führen, dass ich mich suizidal fühle?
* Indirekte Auslöser: Faktoren, die zur Entwicklung von Suizidalität beitragen, aber die nicht zwangsläufig immer sofort zu akuten suizidalen Gedanken und Handlungen führen (z.B. Obdachlosigkeit, Depression, Substanzmissbrauch, traumatische Erfahrungen, Isolation).

____

____

____

____

____

____

____

____

Bestimmte Themen (z.B. Muster in Beziehungen oder im Selbstkonzept)

____

____

____

____

3. Konzeptualisierung der Suizidalität

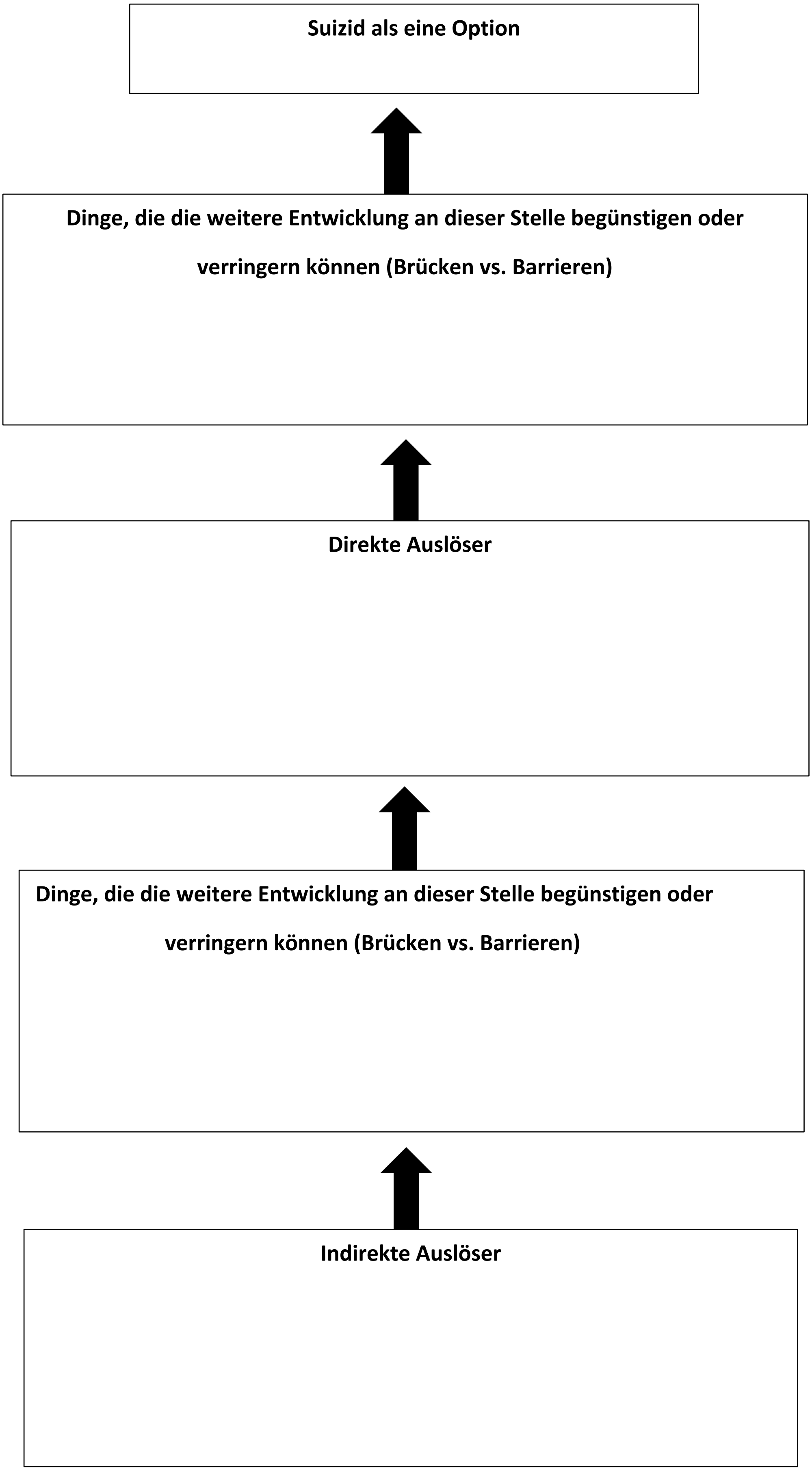

Supplement: Supplementary file 2 — Additional file 2:. Appendix 2 Suicide Status Forms – Complete CAMS Material [file 12888_2020_2589_MOESM2_ESM.pdf]
