# Supplementary material for: Collaborative Assessment and Management of Suicidality (CAMS) compared to enhanced treatment as usual (E-TAU) for suicidal patients in an inpatient setting: study protocol for a randomized controlled trial
Source: BMC Psychiatry. 2020 Apr 22;20:183. doi: 10.1186/s12888-020-02589-x (PMC7178967; doi:10.1186/s12888-020-02589-x)
Supplement: Supplementary file 3 — Additional file 3:. Appendix 3a Einverständniserklärung zur Teilnahme an der Studie – Informed Consent Form [file 12888_2020_2589_MOESM3_ESM.pdf]

EvKB gGmbH I Psychiatrie
Remterweg 69-71 I 33617 Bielefeld

Klinik für Psychiatrie und Psychotherapie | Bethel

Chefarzt: Prof. Dr. Martin Driessen
Remterweg 69-71 (Gilead IV)
D-33617 Bielefeld

Ansprechpartnerin für eventuelle Rückfragen:

Miriam Santel, Dipl.-Psychologin
Psychologische Psychotherapeutin
Telefon: 0521/772-78618
Email: Miriam.Santel@evkb.de

Akademisches Lehrkrankenhaus der Universität Münster

Universitätslehrkrankenhaus der Universität Pécs (Ungarn)

Sitz der Gesellschaft
Kantensiek 11 I 33617 Bielefeld
Zentrale: 0521-772-700

Sparkasse Bielefeld
IBAN DE 6948050161006429655
BIc SPBIDE3BXXX
Amtsgericht Bielefeld HRB 30169

Geschäftsführer
Dr. Rainer Norden

Vorsitzender des Aufsichtsrates
Pastor Ulrich Pohl

Einverständniserklärung zur Teilnahme an der Studie „Behandlung suizidaler Patienten mit dem Collaborative Assessment and Management of Suicidality (CAMS) nach David A. Jobes versus Treatment as Usual (TAU)“

1. Ich habe das Informationsblatt gelesen bzw. vorgelesen bekommen und bin damit einverstanden.

2. Ich wurde von den Mitarbeitern über die Untersuchung aufgeklärt und meine Fragen wurden für mich befriedigend und umfassend beantwortet.

3. Ich bin damit einverstanden, dass im Rahmen des Forschungsvorhabens meine Daten einschließlich der Daten über Gesundheitszustand und Krankengeschichte, Geschlecht und Alter aufgezeichnet und pseudonymisiert[1] (Namen usw. werden durch einen Verschlüsselungscode ersetzt) werden.

4. Ich weiß, dass Daten über meine Person nur anonym[2] verarbeitet werden, und dass alle autorisierten Projektmitarbeiter, die Zugang zu Angaben und Daten zu meiner Person haben, unter Schweigepflicht stehen zu dem Zweck, die Identifizierung des Betroffenen auszuschließen oder wesentlich zu erschweren (§3 Abs. 6 Bundesdatenschutzgesetz).

5. Mir ist bekannt, dass die erhobenen Informationen auf Computern gespeichert und verarbeitet werden, die mit dem Internet verbunden sind.

6. Ich weiß, dass ich in den diagnostischen und psychotherapeutischen Sitzungen zu verschiedenen persönlichen und ggf. belastenden Themen befragt werde und dass es mir im Anschluss möglicherweise vorübergehend schlechter gehen kann. In diesem Falle kann ich mich mit einem Mitarbeiter der Station in Verbindung setzen.

7. Ich nehme freiwillig an der Behandlungsstudie teil und weiß, dass ich die Teilnahme jederzeit abbrechen kann.

---

[1] Pseudonymisieren ist das Ersetzen des Namens und anderer Identifikationsmerkmale durch ein Kennzeichen.
[2] Anonymisieren ist das Verändern personenbezogener Daten derart, dass die Einzelangaben über persönliche oder sachliche Verhältnisse nicht mehr oder nur mit einem unverhältnismäßig großen Aufwand an Zeit, Kosten und Arbeitskraft einer bestimmten oder bestimmbaren natürlichen Person zugeordnet werden können (§3 Abs. 6a Bundesdatenschutzgesetz)

8. Ich bin schriftlich und im persönlichen Gespräch ausreichend über den Inhalt der Studie und die Vor- und Nachteile einer Teilnahme aufgeklärt worden und habe keine weiteren Fragen.

Bielefeld, den ……………………………………………………………

………………………………………………………………

(Unterschrift PatientIn)

……………………………………………………………

(Unterschrift TherapeutIn)

9. Ich erkläre mich bereit, dass Tonbandaufnahmen von meinen therapeutischen Gesprächen gemacht werden. Diese dienen ausschließlich der Supervision der Therapeuten (Supervision ist die qualifizierte Begutachtung und Überwachung der Therapie durch einen erfahrenen unabhängigen Therapeuten).

___ JA ___ NEIN

Bielefeld, den ……………………………………………………………

………………………………………………………………

(Unterschrift PatientIn)

……………………………………………………………

(Unterschrift TherapeutIn)
