# Supplementary material for: Collaborative Assessment and Management of Suicidality (CAMS) compared to enhanced treatment as usual (E-TAU) for suicidal patients in an inpatient setting: study protocol for a randomized controlled trial
Source: BMC Psychiatry. 2020 Apr 22;20:183. doi: 10.1186/s12888-020-02589-x (PMC7178967; doi:10.1186/s12888-020-02589-x)
Supplement: Supplementary file 4 — Additional file 4:. Appendix 3b Informationen zur Studie – Patient information [file 12888_2020_2589_MOESM4_ESM.pdf]

Evangelisches KRANKENHAUS Bielefeld

EvKB gGmbH | Psychiatrie
Remterweg 69/71 | 33617 Bielefeld

Klinik für Psychiatrie und Psychotherapie
Prof. Dr. med. Martin Driessen
Chefarzt
Haus Gilead IV
Remterweg 69/71
33617 Bielefeld

Telefon 05 21 | 7 72-7 84 50
Telefax 05 21 | 7 72-7 84 52
E-Mail martin.driessen@evkb.de
Internet www.evkb.de

Ansprechpartnerin für eventuelle Rückfragen:

Miriam Santel, Dipl.-Psychologin
Psychologische Psychotherapeutin
Telefon: 0521 | 772-78618
Email: Miriam.Santel@evkb.de

Akademisches Lehrkrankenhaus der Universität Münster

Universitätslehrkrankenhaus der Universität Pécs (Ungarn)

Sitz der Gesellschaft
Kantensiek 11 | 33617 Bielefeld
Zentrale: 05 21 | 7 72-7 00

Sparkasse Bielefeld
IBAN DE 69 4805 0161 0006 4296 58
BIC SPBIDE3BXXX
Amtsgericht Bielefeld HRB 30169

Geschäftsführer
Dr. Rainer Norden

Vorsitzender des Aufsichtsrates
Pastor Ulrich Pohl

Informationen zur Studie „Behandlung suizidaler Patienten mittels des Collaborative Assessment and Management of Suicidality (CAMS) nach David A. Jobes versus Treatment as Usual (TAU)“

Sehr geehrte Patientin, sehr geehrter Patient,

vielen Dank für Ihr Interesse an unserer Studie. Wir bitten Sie, diese Informationen sorgfältig zu lesen und anschließend zu entscheiden, ob Sie an dieser Behandlung und Untersuchung teilnehmen möchten. Für weitere Fragen steht Ihnen Ihr Therapeut selbstverständlich gern zur Verfügung.

Was sind die Ziele der Studie?

Das Ziel dieser Studie ist es, die Behandlung von Menschen mit lebensmüden Gedanken oder nach bereits erfolgten Suizidversuchen zu verbessen. Wir erhoffen uns, Ihre Gründe und Auslöser für suizidale Tendenzen besser zu verstehen und herauszufinden, welche Inhalte Sie im Rahmen unserer Behandlung als hilfreich erleben.

Das „Collaborative Assessment and Management of Suicidality“ (CAMS) ist ein Suizidpräventionsansatz, der von Herrn David A. Jobes in den USA entwickelt und in den vergangenen 25 Jahren regelmäßig weiterentwickelt wurde, um den Herausforderungen in der Behandlung von Patienten mit Suizidgedanken angemessen zu begegnen. Im Rahmen des CAMS sollen mit Hilfe eines strukturierten Vorgehens die Ursachen und Gründe Ihrer Suizidalität genau verstanden und daraus Veränderungen abgeleitet werden.

Mit Hilfe dieser Untersuchung soll die Wirksamkeit des CAMS für die Behandlung von suizidalen Patienten überprüft werden. Die bisherigen Forschungsergebnisse sind sehr vielversprechend: Es gibt erste Studien, die darauf hinweisen, dass das CAMS suizidale Gedanken und die Belastung durch andere Symptome rasch und anhaltend verringert.
Neben dem CAMS untersuchen wir unsere reguläre therapeutische Behandlung (Treatment As Usual, TAU). Auch im TAU steht die Reduzierung Ihrer suizidalen Gedanken und Belastungen im Vordergrund, doch unterliegt diese Behandlung keiner vorgegeben Struktur, sondern lässt Ihnen und Ihrem Therapeuten entsprechenden Freiraum in der Auswahl von Gesprächsinhalten.
Um noch verbleibende Fragen zur Wirksamkeit des CAMS und unserer regulären Behandlung zu klären, möchten wir **zu Beginn**, **während** und **nach** der Behandlung Ihre Meinung und Ihre derzeitige Belastung durch Symptome anhand von Fragebögen erheben (Dauer ca. 20 Minuten). Um die Wirksamkeit der Behandlungen ohne Verzerrungen feststellen zu können und Verfälschungen vorzubeugen**, nehmen wir eine randomisierte (d.h. zufällige) Zuteilung zu den beiden Therapieformen (TAU und CAMS) vor.**

Wie ist der organisatorische Ablauf der Studie?

Im Rahmen des Aufnahmegesprächs überprüft Ihr Therapeut die Teilnahmevoraussetzungen für unsere Studie. Wenn feststeht, dass unsere Therapieformen für Sie geeignet sind, können Sie an unserer Studie teilnehmen. Es wird dann per Zufall entschieden, ob Sie mit der CAMS- oder der TAU-Behandlung beginnen. Beide Verfahren bringen eine stationäre Behandlungsdauer von ca. 10 bis 20 Tagen mit sich. In dieser Zeit erhalten Sie mindestens drei 45 bis 60-minütige therapeutische Gespräche. Neben dem jeweils angewandten Therapiekonzept erhalten Sie zusätzliche Therapieangebote der Station. Nach der Entlassung aus der stationären Behandlung bieten wir Ihnen ca. 4 Wochen später ein weiteres therapeutisches Gespräch an, und es erfolgt eine Nachuntersuchung. Etwa 5 Monate nach der Behandlung erhalten Sie per Post erneut Fragebögen, anhand derer wir die langfristige Wirksamkeit Ihrer Behandlung untersuchen.

Welche Risiken und Vorteile gibt es?

Es gibt keine bekannten Risiken, die mit der Teilnahme an dieser Studie verbunden sind. Wenn Sie sich durch die Teilnahme an unserer Untersuchung in irgendeiner Weise belastet fühlen, steht Ihnen das Behandlungsteam der Station hierfür selbstverständlich zur Seite. Im Rahmen der Studie bieten wir Ihnen eine kostenlose, ausführliche Diagnostik an und begleiten Sie sehr engmaschig. Sie haben eventuell die Möglichkeit ein neues Therapieverfahren auszuprobieren, welches in der gewöhnlichen Versorgung von Patienten nicht angeboten wird und sich positiv auf Ihre suizidalen Gedanken sowie Ihre Belastung durch andere Symptome und ihre Lebensqualität auswirken könnte. Ergänzend zu den regelmäßigen therapeutischen Gesprächen, steht Ihnen ein weiteres persönliches Gespräch 4 Wochen nach der stationären Behandlung zu. Darüber hinaus erhalten Sie für das vollständige Bearbeiten der Fragebögen 4 Wochen und 5 Monate nach der Behandlung jeweils 10 Euro als Aufwandsentschädigung.

Wie sind die Teilnahmebedingungen und wie wird mit persönlichen Daten umgegangen?

Die Teilnahme an der Studie ist **freiwillig**. Sie können jederzeit und ohne Angabe von Gründen Ihre Einwilligung zur Teilnahme widerrufen, ohne dass Ihnen daraus Nachteile entstehen. Auch wenn Sie die Studienteilnahme vorzeitig abbrechen, haben Sie weiterhin die Möglichkeit einer regulären Behandlung in unserer Klinik, soweit dies als sinnvoll erscheint.
Wenn Sie einverstanden sind, werden die Behandlungssitzungen zur Qualitätssicherung auf Tonband aufgenommen. Anhand dieser Aufnahmen wird die Qualität der Therapie von einem besonders erfahrenen Kollegen analysiert und zur Verbesserung der Behandlung ggf. mit Ihrem Therapeuten besprochen. Alle Personen, die Zugang zu diesen Aufzeichnungen haben, unterliegen der Schweigepflicht entsprechend § 203 StGB. Die Tonbandaufnahmen werden unmittelbar nach der Auswertung vernichtet.
Die Erhebung der Fragebogendaten erfolgt pseudonymisiert, d.h. in namentlich nicht gekennzeichneter Form. Die Pseudonymisierung Ihrer Daten erfolgt durch die Vergabe von fortlaufenden Code-Nummern. Nur autorisierte Mitarbeiter des Forschungsprojektes haben Zugang zu den erhobenen Daten. Die pseudonymisierten Daten werden in der Forschungsabteilung archiviert und nach zehn Jahren sowie bei Widerruf der Einwilligung vernichtet. Die Ergebnisse der Studie können als wissenschaftliche Publikationen veröffentlicht werden. Dies geschieht in anonymisierter Form, d.h. ohne dass Ihre Daten Ihrer Person zugeordnet werden können.
Sollten Sie die Informationsweitergabe an Dritte wünschen, so geschieht dies ausschließlich auf Ihre schriftliche Veranlassung und eine Schweigepflichtentbindung hin.

Herzlichen Dank für Ihr Interesse!

Projektkoordination und Ansprechpartnerin:

Miriam Santel
Telefon: 0521/772-78618
