# Supplementary material for: Collaborative Assessment and Management of Suicidality (CAMS) compared to enhanced treatment as usual (E-TAU) for suicidal patients in an inpatient setting: study protocol for a randomized controlled trial
Source: BMC Psychiatry. 2020 Apr 22;20:183. doi: 10.1186/s12888-020-02589-x (PMC7178967; doi:10.1186/s12888-020-02589-x)
Supplement: Supplementary file 6 — Additional file 6:. Appendix 5a Ethikkommission; Beratung und Bewertung – Consent of the Ethical Committee: Original Document in German [file 12888_2020_2589_MOESM6_ESM.pdf]

Ø Bebko Ø Santel

Patientenschutz | Forschungsfreiheit

ÄRZTEKAMMER WESTFALEN-LIPPE

WESTFÄLISCHE WILHELMS-UNIVERSITÄT MÜNSTER

ETHIK KOMMISSION
der Ärztekammer Westfalen-Lippe und der Westfälischen Wilhelms-Universität

EV. KLINIKUM Bethel gGmbH
Eingang
Dat. 10. März 2017
Klinik für Psychiatrie und Psychotherapie

Gartenstraße 210 – 214
48147 Münster, Germany
Tel.: +49 (0)251 929 2460
Fax: +49 (0)251 929 2478
E-Mail: ethik-kommission@aekwl.de
www.ethik-kommission.uni-muenster.de

Ethik-Kommission Münster · Gartenstraße 210 – 214 · 48147 Münster

Herrn
Prof. Dr. med. Martin Driessen
Klinik für Psychiatrie u. Psychotherapie in Bethel (KPPB)
Evangelisches Krankenhaus Bielefeld gGmbH
Remterweg 69 - 71 (Gilead IV)
33617 Bielefeld

6. März 2017

**Unser Aktenzeichen:**	**2016-620-f-S** (bitte immer angeben!)
Studiencode:	CAMS versus TAU
Sponsor / Finanzierung:	Evangelisches Krankenhaus Bielefeld gGmbH, Bielefeld
Titel des Forschungsvorhabens:	„Evaluation von CAMS versus TAU bei suizidalen Patienten - Ein stationärer RCT CAMS = Collaborative Assessment and Management of Suicidality"

Beratung und Bewertung

Sehr geehrter Herr Professor Driessen,

für das oben genannte Forschungsvorhaben haben Sie mit Schreiben vom 15.11.2016 die Beratung durch die Ethik-Kommission der Ärztekammer Westfalen-Lippe und der Westfälischen Wilhelms-Universität Münster („Ethik-Kommission") beantragt. Die Ethik-Kommission hat in ihrer Sitzung am 13.01.2017 über Ihren Antrag beraten, ergänzend vorgelegte Unterlagen in einem Ausschuss nach § 5 Abs. 1 Satz 3 ihrer Satzung geprüft und beschlossen:

Die Ethik-Kommission hat keine grundsätzlichen Bedenken ethischer oder rechtlicher Art gegen die Durchführung des Forschungsvorhabens.

Die vorliegende Einschätzung gilt für das Forschungsvorhaben, wie es sich auf Grundlage der in Anhang 1 genannten Unterlagen darstellt.

Für die Entscheidung der Ethik-Kommission erhebt die Ärztekammer Westfalen-Lippe Gebühren nach Maßgabe ihrer Verwaltungsgebührenordnung. Über die Gebühren erhalten Sie von der Ärztekammer einen gesonderten Bescheid.

Allgemeine Hinweise:

Mit der vorliegenden Stellungnahme berät die Ethik-Kommission die der Ärztekammer Westfalen-Lippe angehörenden Ärztinnen und Ärzte zu den mit dem Forschungsvorhaben verbundenen berufsethischen und berufsrechtlichen Fragen gemäß § 15 Abs. 1 Berufsordnung ÄKWL.
Die Einschätzung der Kommission ist als ergebnisoffene Beratung für den Antragsteller nicht bindend. Die Ethik-Kommission weist darauf hin, dass unabhängig von der vorliegenden Stellungnahme die medizinische, ethische und rechtliche Verantwortung für die Durchführung des Forschungsvorhabens bei dessen Leiter und bei allen an dem Vorhaben teilnehmenden Ärzten bzw. Forschern verbleibt.

An der Beratung und Beschlussfassung haben die in Anhang 2 aufgeführten Mitglieder der Ethik-Kommission teilgenommen. Es haben keine Mitglieder teilgenommen, die selbst an dem Forschungsvorhaben mitwirken oder deren Interessen davon berührt werden.

Die Ethik-Kommission empfiehlt im Einklang mit der Deklaration von Helsinki nachdrücklich die Registrierung klinischer Studien vor Studienbeginn in einem öffentlich zugänglichen Register, das die von der Weltgesundheitsorganisation (WHO) geforderten Voraussetzungen erfüllt, insbesondere deren Mindestangaben enthält. Ausführliche Informationen zur International Clinical Trials Registry Platform (ICTRP) stehen im Internetangebot der WHO zur Verfügung:

http://www.who.int/ictrp/about/en/

Zu den Kriterien des International Committee of Medical Journal Editors (ICMJE) sei beispielsweise verwiesen auf die Informationen unter:

http://www.icmje.org/recommendations/browse/publishing-and-editorial-issues/clinical-trial-registration.html

Das WHO Primär-Register für Deutschland ist das Deutsche Register für Klinische Studien (DRKS) in Freiburg. Es erfüllt die Forderungen der Fachzeitschriften:

http://www.drks.de/index.html

Die Ethik-Kommission der Ärztekammer Westfalen-Lippe und der Westfälischen Wilhelms-Universität Münster ist organisiert und arbeitet gemäß den nationalen gesetzlichen Bestimmungen und den GCP-Richtlinien der ICH.

Die Kommission wünscht Ihrem Forschungsvorhaben gutes Gelingen und geht davon aus, dass Sie nach Abschluss des Vorhabens über die Ergebnisse berichten werden.

Mit freundlichen Grüßen

Univ.-Prof. Dr. med. Hans-Werner Bothe M.A.
Vorsitzender der Ethik-Kommission

Anhang 1

Folgende Unterlagen haben bei der Beschlussfassung vorgelegen:
Bei mehreren Versionen eines Dokumentes bezieht sich unsere Bewertung stets auf die letzte Version.

Eingang	Datierung	Anlage
21.11.2016	15.11.2016	3_CAMS_Fragebogenpaket Ethik
21.11.2016	15.11.2016	2_CAMS_Studienmaterial Ethik
21.11.2016	15.11.2016	1_CAMS_Ethikantrag
27.12.2016	22.12.2016	CAMS-Ethikantrag-Formular-20.12.2016
27.12.2016	22.12.2016	CAMS-Studienmaterial Ethik
27.12.2016	22.12.2016	Fragebogenpaket CAMS Ethik
27.12.2016	22.12.2016	CAMS-Studienmaterial gesamt
13.02.2017	07.02.2017	Einverständniserklärung CAMS-Studie
13.02.2017	07.02.2017	Patienteninformationen zur CAMS-Studie
13.02.2017	07.02.2017	Antwort auf Nachfrage

Anhang 2

Folgende Mitglieder der federführenden Ethik-Kommission haben an der abschließenden Beratung und Beschlussfassung in der Sitzung vom 13.01.2017 teilgenommen:

Prof. Dr. med.
Peter **Baumgart**
Clemenshospital GmbH, Klinik für Innere Medizin I

Dr. med. M.A., LL.M.
Doris **Dorsel**
Ärztekammer Westfalen-Lippe

Univ.-Prof. Dr. med.
Frank Ulrich **Müller**
Universitätsklinikum Münster, Institut für Pharmakologie und Toxikologie

Univ.-Prof. Dr. jur.
Heinz-Dietrich **Steinmeyer**
Westfälische Wilhelms-Universität Münster, Institut für Arbeits-,Sozial- und Wirtschaftsrecht II

Prof. Dr. med.
Constantin **Uhlig**
Universitätsklinikum Münster, Klinik für Augenheilkunde

Univ.-Prof. Dr. Dr. med. M.A. phil.
Hans-Werner **Bothe**
Universitätsklinikum Münster, Experimentelle Neurochirurgie

Univ.-Prof. Dr.
Wolfgang **Köpcke**
Westfälische Wilhelms-Universität Münster, Institut für Biometrie und klinische Forschung

Prof. Dr. med.
Heinrich **Schulze-Mönking**
St. Rochus-Hospital Telgte GmbH, Abt. für Psychiatrie und Psychotherapie

Alfred **Storck**
Sonderschulrektor i.E., St. Elisabeth Schule, Steinfurt

Dr. rer. nat.
Dorothea **Voß**
Apothekerin für klinische Pharmazie und Apothekerin für prakt. und theoret. Ausbildung i.R., Münster
