# Supplementary material for: Collaborative Assessment and Management of Suicidality (CAMS) compared to enhanced treatment as usual (E-TAU) for suicidal patients in an inpatient setting: study protocol for a randomized controlled trial
Source: BMC Psychiatry. 2020 Apr 22;20:183. doi: 10.1186/s12888-020-02589-x (PMC7178967; doi:10.1186/s12888-020-02589-x)
Supplement: Supplementary file 7 — Additional file 7:. Appendix 5b Ethikkommission; Consulting and Evaluation – Consent of the Ethical Committee: English translation [file 12888_2020_2589_MOESM7_ESM.pdf]

Ø Betto Ø S .

Patientenschutz | Forschunqsfrelheit

ÄRZTEKAMMER WESIEALEN-LIFTE

ETHIKINI KOMMISSION

Der Ärztekammer Westfalen-Lippe und der Westfälischen Wilhelms-Universität

EV. KLINIKU
Dat. "9 gGmbH
und Psychotherapie

Gartenstraße 210-214
48147 Münster, German/
701.: +49 (0)251 929 2460
Fax: +49 (0)251 929 2478
E-Mail: ethlk-kommlsslonftaekx+1 de
www.ethlk-kommlssion.unl-muensterde

6. March 2017

Ethik-Kommission Münster Gartenstraße 210 - 214 411147 Münster

Herrn
Prof. Dr. med. Martin Driessen
Klinik für Psychiatrie u. Psychotherapie in Bethel (KPPB)
Evangelisches Krankenhaus Bielefeld gGmbH
Remterweg 69 - 71 (Gilead IV)
33617 Bielefeld

**Our file number:**	**2016-620-f-S** (please always specify!)
Study code:	CAMS versus TAU
Sponsor / Funding:	Evangelisches Krankenhaus Bielefeld gGmbH, Bielefeld
Title of the Research Project:	„Evaluation of CAMS versus TAU with suicidal patients - An inpatient RCT CAMS = Collaborative Assessment and Management of Suicidality"

Consulting and Evaluation

Dear Professor Driessen,

for the above-mentioned research project, you have applied for advice from the Ethics Commission of the Ärztekammer Westfalen-Lippe and the Westfälische Wilhelms Universität Münster ("Ethics Commission") in your letter of 15.11.2016. At its meeting on 13.01.2017, the Ethics Commission discussed your application, examined and decided on the additional documents submitted in a committee in accordance with § 5 para. 1 sentence 3 of its statutes:

The Ethics Committee has no fundamental ethical or legal concerns for the implementation of the research project.

The present assessment applies to the research project as presented on the basis of the documents referred to in Annex 1.

The Ärztekammer Westfalen-Lippe charges fees for the decision of the Ethics Committee in accordance with its administrative fee schedule. You will receive a separate notification of the fees from the Ärztekammer.

General information:

With the present statement, the Ethics Committee advises the physicians belonging to the Ärztekammer Westfalen-Lippe on the ethical and legal issues associated with the research project in accordance with § 15 para. 1 of the ÄKWL professional code of conduct. The Commission's assessment is not binding for the applicant as an open-ended consultation. The Ethics Committee points out that, irrespective of the present statement, the medical, ethical and legal responsibility for the implementation of the research project remains with its director and all physicians or researchers participating in the project.

Vorsitzender Univ-Prof. Dr Dr med, 1-1 -W Bothe M A phil
Stellvertretende Vorsitzende Univ -Prof Dr rned W r Borde', Prof Dr phil C Frantz, Univ-Prof Dr med 1- 11 Müller

The members of the Ethics Committee listed in Appendix 2 took part in the deliberations and decision-making process. No members participated who were themselves involved in the research project or whose interests were affected by it.

In accordance with the Declaration of Helsinki, the Ethics Committee strongly recommends that clinical trials be registered before the start of studies in a publicly accessible register that meets the requirements of the World Health Organization (WHO), in particular the minimum information required. Detailed information on the International Clinical Trials Registry Platform (ICTRP) is available on the WHO website:

http://www.who.int/ictrp/about/en/

For the criteria of the International Committee of Medical Journal Editors (ICMJE), for example, reference is made to the information below:

http://www.icmje.org/recommendations/browse/publishing-and-editorial-issues/clinical-trial-registration.html

The WHO Primary Registry for Germany is the German Registry for Clinical Trials (DRKS) in Freiburg. It meets the requirements of the scientific journals:

http://www.drks.de/index.html

The Ethics Commission of the Westphalia-Lippe Medical Association and the Westphalian Wilhelms University of Münster is organised and operates in accordance with the national regulations and the GCP guidelines of the ICH.

The Commission wishes your research project every success and assumes that you will report on the results once the project has been completed.

Yours sincerely

Univ.-Prof. Dr. med Hans-Werner Bothe M.A.
Vorsitzender der Ethik-Kommission

Anhang 1

The following documents were available at the time the resolution was adopted:
In the case of several versions of a document, our evaluation always refers to the latest version of the document version.

**Inbox**	**Dating**	**Apendix**
21.11.2016	15.11.2016	3_CAMS_Questionnaires Ethics
21.11.2016	15.11.2016	2_CAMS_Study Material Ethics
21.11.2016	15.11.2016	1_CAMS_Ethical Approval Document
27.12.2016	22.12.2016	CAMS-Ethical Approval Document Form-20.12.2016
27.12.2016	22.12.2016	CAMS-Material Ethics
27.12.2016	22.12.2016	Questionnaires CAMS Ethics
27.12.2016	22.12.2016	CAMS-Stud Material total
13.02.2017	07.02.2017	Consent form CAMS study
13.02.2017	07.02.2017	Patient information to the CAMS study
13.02.2017	07.02.2017	Answer to inquiry

Anhang 2

The following members of the leading ethics committee took part in the final consultation and resolution in the meeting of 13.01.2017:

Prof. Dr. med.
Peter **Baumgart**
Clemenshospital GmbH, Klinik für Innere Medizin I

Dr. med. M.A., LL.M.
Doris **Dorsel**
Ärztekammer Westfalen-Lippe

Univ.-Prof. Dr. med.
Frank Ulrich **Müller**
Universitätsklinikum Münster, Institut für Pharmakologie und Toxikologie

Univ.-Prof. Dr. jur.
Heinz-Dietrich **Steinmeyer**
Westfälische Wilhelms-Universität Münster, Institut für Arbeits-,Sozial- und Wirtschaftsrecht II

Prof. Dr. med. Constantin **Uhlig**
Universitätsklinikum Münster
Klinik für Augenheilkunde

Univ.-Prof. Dr. Dr. med. M.A. phil.
Hans-Werner **Bothe**
Universitätsklinikum Münster, Experimentelle Neurochirurgie

Univ.-Prof. Dr.
Wolfgang **Köpcke**
Westfälische Wilhelms-Universität Münster, Institut für Biometrie und klinische Forschung

Prof. Dr. med.
Heinrich **Schulze-Mönking**
St. Rochus-Hospital Telgte GmbH, Abt. für Psychiatrie und Psychotherapie

Alfred **Storck**
Sonderschulrektor i.E., St. Elisabeth Schule, Steinfurt

Dr. rer. nat.
Dorothea **Voß**
Apothekerin für klinische Pharmazie und Apothekerin für praktische und theoretische Ausbildung
i.R., Münster
